# Research on the Changing Characteristics of Milk Composition and Serum Metabolites Across Various Lactation Periods in Xinggao Sheep

**DOI:** 10.3390/metabo15100678

**Published:** 2025-10-20

**Authors:** Jingda Yuan, Zhenbo Wu, Biao Wang, Shaoyin Fu, Rigele Te, Lai Da, Liwei Wang, Qing Qin, Xiaolong He

**Affiliations:** 1 Inner Mongolia Academy of Agricultural & Animal Husbandry Sciences, Hohhot 010031, China; yuanjingda0825@163.com (J.Y.); wb@imaaahs.ac.cn (B.W.); fsy@imaaahs.ac.cn (S.F.); terigele-2009@163.com (R.T.); 18747977760@163.com (L.W.);; 2 National and Local Joint Engineering Research Center for Genetic Resource Evaluation and Breeding Technology of Meat Sheep, Hohhot 010031, China; 3 College of Animal Science, Inner Mongolia Agricultural University, Hohhot 010018, China

**Keywords:** lactating ewes, intensive feeding, serum metabolites, milk components

## Abstract

Background: The variation in sheep milk composition is closely related to the sheep’s metabolic status. This study aimed to analyze the milk composition and serum metabolic characteristics of Xinggao sheep during different lactation periods and to evaluate the association between milk quality traits and body metabolism. Methods: Eighteen intensively reared ewes were divided into three groups: an early lactation group (MA), a mid-lactation group (MB), and a late lactation group (MC). Milk components were detected by infrared spectroscopy, and the ewes’ serum metabolomic characteristics were detected by liquid chromatography–mass spectrometry (LC-MS). K-means correlation analysis revealed that the milk fat percentage was positively correlated with L-aspartic acid and negatively correlated with citrulline levels. Random forest analysis for metabolite importance ranking showed that methionine sulfoxide and methionine exhibited high mean decrease accuracy and mean decrease Gini index values. Results: The milk composition results showed that, compared with MA, the milk fat content and total solids in MB and MC were significantly higher, while the freezing point in the MC was significantly lower. Metabolomic studies showed that 207, 210, and 238 differential metabolites were identified in the comparisons of MA vs. MB MA vs. MC, and MB vs. MC, respectively, and these metabolites were mainly enriched in the pyrimidine metabolism, arachidonic acid metabolism, and arginine biosynthesis pathways. Evaluation of metabolite importance using random forest models revealed that 27 metabolites, including 2-Arachidonyl glycerol ether, methionine, and methionine sulfoxide, showed a high mean decrease accuracy and mean decrease Gini index. Correlation analysis revealed that milk fat percentage and total solids were positively correlated with 11 metabolites, including citrulline, phenylalanine, and octadecylamine, and negatively correlated with isoproterenol, cortisol, and kynurenic acid. The freezing point was positively correlated with cortisol, isoproterenol, and kynurenic acid and negatively correlated with aldosterone, dehydroepiandrosterone, and betaine. Conclusions: This study showed that there were significant differences in the milk composition and metabolites of Xinggao sheep during different lactation periods, highlighting the impact of lactation stage on milk composition and production performance. We recommend developing targeted nutritional strategies based on the specific metabolic profiles of different lactation periods to optimize the feeding management and nutritional regulation of Xinggao sheep.

## 1. Introduction

As the optimal nutritional source for lambs, breast milk provides irreplaceable nutritional, growth, and immune support for lambs through its unique composition [1]. The dynamic changes in milk composition directly affect the growth and development of lambs, and these changes exhibit complex physicochemical characteristics due to the lactation stage [2], breed [3], and sheep management practices [4]. In modern intensive farming, early weaning strategies are widely adopted to improve the reproductive efficiency of ewes, including enhancing their conception rate, lambing rate, and offspring birth weight [5]. To better manage this process, the lactation period is typically divided into three stages: early, middle, and late. Considering the crucial role of breast milk in lamb development, in-depth analysis of changes in milk composition and underlying metabolic mechanisms of ewes at different lactation stages can not only optimize early weaning strategies and lamb rearing programs but also provide a scientific basis for improving the economic benefits of intensive farming.

In recent years, researchers have systematically analyzed the molecular regulatory networks during lactation cycles in species such as dairy cattle and goats through various approaches [6,7,8]. However, comparatively speaking, lactation research in sheep has lagged behind, mainly focusing on the physicochemical properties of milk components or mechanistic studies of single components (such as whey change mechanisms), with research on the association between milk composition and metabolic changes in ewes under intensive feeding systems being particularly scarce, constraining precise sheep management. Ewes undergo significant changes in metabolic status and milk composition at different lactation stages, and these changes are closely correlated. This is because blood contains precursor substances of milk components, which not only transport various substances through the circulatory system but also carry nutrients after digestion and absorption [9]. These circulating substances and absorbed nutrients work together, ultimately leading to macroscopic changes in milk composition.

Metabolomics research helps identify and predict biomarkers or metabolic pathways for biological responses or health conditions, and the identification and measurement of metabolites can provide key insights into understanding overall physiological processes [10]. For example, Rempel et al. [11] compared sows at different lactation periods through metabolomics and found that caprolactam may indirectly promote uterine and ovarian mobilization, preparing for the next reproduction after lactation ends. Fu et al. [12] also conducted metabolic comparisons of dairy cows at different lactation periods using metabolomics technology and found that cows at peak lactation regulate body energy balance through adaptive adjustment of branched-chain amino acid levels. These studies demonstrate the tremendous potential of metabolomics in revealing complex physiological mechanisms during lactation. Therefore, this study compares changes in the milk composition and serum metabolic profiles of intensively raised ewes at different lactation stages to reveal the macroscopic effects and potential associations of body metabolism on milk composition changes during different lactation periods, aiming to provide scientific basis for optimizing flock management and improving overall production efficiency.

## 2. Materials and Methods

### 2.1. Experimental Design and Animals

The animal experiments conducted in this study received approval from the Animal Care and Use Committee at the Inner Mongolia Academy of Agricultural and Animal Husbandry Sciences. This experiment was conducted from 4 December 2023 to 10 January 2024. Six housed Xinggao sheep of a similar age and parity, with all being little in size and also similar in body weight (42.3 ± 3.46 kg), were selected from Inner Mongolia Dumei Animal Husbandry Biotechnology Co., Ltd. in Jalaid Banner, Inner Mongolia, China, for each of the three lactation stages—early lactation (7 ± 3 days postpartum; MA), mid-lactation (25 ± 3 days; MB), and late lactation (40 ± 3 days; MC)—for blood and milk sample collection. The feeding and management mode of the lactating sheep was implemented completely according to the actual feeding and management mode of the sheep farm.

### 2.2. Sample Collection and Index Determination

#### 2.2.1. Milk Composition Analysis

Milk samples were collected from lactating ewes in the morning and evening on the collection day, with a total of 50 mL collected. To eliminate contamination, 20 mL of milk was first discarded, and then fresh milk samples were collected into sterile cryovials. Morning and evening milk samples were mixed in equal proportions, with bronopol added as a preservative. Subsequently, samples were stored at 4 °C and sent to the Inner Mongolia Academy of Agricultural and Animal Husbandry Sciences, where main components (milk fat rate, milk protein rate, lactose, non-fat milk solids, density, freezing point, acidity, and total solids) were detected using a FossFT120 milk composition analyzer (FOSS A/S, Hillerød, Denmark). The analytical method followed the NY/T 2659-2014 [13] infrared spectroscopy analysis standard. K-means analysis was then employed to perform cluster analysis on the variation trends of milk components, followed by Pearson correlation analysis.

#### 2.2.2. Blood Sample Collection and Serum Metabolomics Determination

On the morning of milk sample collection, 5 mL of blood was collected from the jugular vein of fasting ewes before feeding and allowed to stand for 20 min, and serum was separated using a centrifuge (3000 r/min, 5 min) and stored at −20 °C for testing. In addition, 100 μL of serum sample was mixed with 400 μL of pre-chilled 80% methanol and thoroughly vortexed. Then, the samples were incubated on ice for 5 min and centrifuged at 15,000× *g*, 4 °C for 20 min. The supernatant was diluted to a final concentration containing 53% methanol by LC-MS grade water. The samples were subsequently transferred to a fresh Eppendorf tube and then were centrifuged at 15,000× *g*, 4 °C for 20 min. Finally, the supernatant was collected and injected into the LC-MS/MS system for analysis, with an injection volume of 5 μL for positive-ion mode and 10 μL for negative-ion mode.

Ultra-high-performance liquid chromatography–tandem mass spectrometry (UHPLC–MS/MS) analysis employed a Vanquish UHPLC system (Thermo Fisher Scientific, Bremen, Germany) coupled to an Orbitrap Q Exactive™ HF-X mass spectrometer (Thermo Fisher Scientific, Bremen, Germany). The autosampler/sample tray temperature was set at 4 °C. Samples were injected onto a Hypersil Gold C18 column (100 × 2.1 mm, 1.9 μm, stationary phase: octadecylsilane (ODS)) with the column temperature set at 40 °C, and eluted with a linear gradient at a flow rate of 0.2 mL/min, with a total run time (including re-equilibration time) of 12 min. The eluents for positive and negative polarity modes were phase A (water containing 0.1% formic acid) and phase B (methanol), respectively. The solvent gradient was programmed as follows: 0–1 min, 2% B; 1–4 min, B increased from 2% to 85%; 4–10 min, B increased from 85% to 100%; 10–10.1 min, B decreased from 100% to 2%; 10.1–12 min, B held at 2% (consistent with total run time). The Orbitrap Q Exactive™ HF mass spectrometer was operated in positive/negative polarity modes with an ESI (electrospray ionization) source, spray voltage of 3.5 kV, capillary temperature of 320 °C, sheath gas at 35 psi, auxiliary gas flow at 10 L/min, S-lens RF level of 60, auxiliary gas heater temperature of 350 °C, and scan range of m/z 100–1500. For MS/MS parameters: the first-order scan (MS1) resolution was 60,000 fwhm, with an automatic gain control (AGC) target of 3 × 10^6^ and a maximum injection time of 100 ms; the second-order scan (MS2) resolution was 15,000 fwhm, with an AGC target of 2 × 10^5^ and a maximum injection time of 25 ms. The MS/MS secondary scan adopted a data-dependent scan (DDA) acquisition mode, with collision energies applied in three steps—20 V, 40 V, and 60 V—to obtain comprehensive fragment ion information.

The raw data files acquired via UHPLC-MS/MS were subjected to processing using Compound Discoverer 3.3 (CD3.3, ThermoFisher), with the aim of conducting peak alignment (mass deviation tolerance: 10 ppm; retention time deviation tolerance: 2 min), peak picking, and quantitation for each metabolite. The key parameters were configured as follows: peak area was calibrated using the first quality control (QC) sample; the actual mass tolerance was set at 5 ppm; the signal intensity tolerance was 30%; and parameters such as minimum intensity were included, among others. Following this step, peak intensities were normalized against the total spectral intensity. After normalization, a Blank sample was used for background interference removal: metabolites with peak area in the Blank sample greater than that in QC1 were eliminated. The normalized data were then utilized to infer the molecular formula based on adduct ions, molecular ion peaks, and fragment ions. Subsequently, the peaks were matched against the mzCloud (https://www.mzcloud.org/ (accessed on 12 June 2024)), mzVault, and MassList databases to obtain accurate qualitative results and relative quantitative data. Statistical analyses were carried out using the statistical software packages R (version R-3.4.3), Python (version 2.7.6), and CentOS (release 6.6). In cases where the data exhibited a non-normal distribution, standardization was performed using the following formula: (raw quantitation value of the sample)/[(sum of quantitation values of all metabolites in the sample)/(sum of quantitation values of all metabolites in the first QC (QC1) sample)], so as to derive relative peak areas. For missing value handling: metabolites with missing value ratios exceeding 50% in all experimental samples (excluding QC samples) were removed from the dataset; the K-nearest neighbor (KNN) algorithm was used to impute the remaining missing values. Compounds for which the coefficient of variation (CV) of relative peak areas in QC samples exceeded 30% were excluded. In addition, metabolite deduplication was performed: first, deduplication was based on InChIKey; if InChIKeys were the same, the compound with smaller level value was retained, and if level values were also the same, the compound with the highest score was retained; for compounds without InChIKey, deduplication was conducted using English names, following the same retention rules as above. All experimental samples were analyzed in the same batch to avoid inter-batch systematic errors, and QC samples were inserted between experimental samples to evaluate the system stability throughout the experiment and conduct data quality control analysis. Regarding injection order: samples were injected one by one in the order of dispatch; before each batch of detection, blank solvent was injected three times to equilibrate the chromatographic column and mass spectrometry system. Ultimately, the results pertaining to the identification and relative quantitation of metabolites were obtained.

Normalized LC-MS data were subjected to multivariate statistical analysis using SIMCA-P14.1 software, including unsupervised principal component analysis (PCA) and supervised partial least squares discriminant analysis (PLS-DA), to explore metabolite profile variation patterns between groups. Generate a scree plot to depict the variance-explained trend, analyze the contribution of each PC to the total variance in the PCA plot, and evaluate the effectiveness of the core components retained for dimensionality reduction. PLS-DA model evaluation was based on R2X and R2Y parameters (representing the degree of explanation for X and Y variables, respectively) and the Q2 value (reflecting the model’s predictive ability). The screening criteria for differential metabolites (DMs) were as follows: variable importance in projection (VIP) value greater than 1 in the PLS-DA model and a *p*-value less than 0.05 in Student’s *t*-test. Fold change (FC) values between the two groups were calculated simultaneously. To investigate metabolic pathway involvement, this study subjected differential metabolites to additional functional annotation using the Kyoto Encyclopedia of Genes and Genomes (KEGG, https://www.kegg.jp/ (accessed on 13 June 2024)). This study’s assignment of differential metabolites to KEGG pathways yielded enrichment analysis outcomes that facilitate mechanistic investigation. Additionally, a primary classification proportion circle diagram was drawn for the differential metabolites. Random forest analysis was performed on differential metabolites, with metabolomic variable data input as feature variables to construct a classifier. Through two feature importance evaluation indicators—mean decrease accuracy and mean decrease Gini index—the influence degree of each feature variable on classification results was measured to screen out key indicators for classifying different lactation stages of Xinggao sheep.

### 2.3. Statistical Analysis

SPSS 19.0 was used to perform one-way analysis of variance (ANOVA) on milk composition data. The homogeneity of variances was assessed with Levene’s test; normality was evaluated with the Shapiro–Wilk test. If both homoscedasticity and normality were satisfied, post hoc multiple comparisons were performed using Tukey’s test; if the data deviated from normality, analyses were conducted using the bootstrap method with 1000 resamples, and confidence intervals were computed with the bias-corrected and accelerated (BCa) approach. Statistical significance was set at *p* < 0.05, and high significance was established at *p* < 0.01. For the statistical analysis of metabolites, statistical analyses were carried out using the statistical software packages R (version R-3.4.3), Python (version 2.7.6), and CentOS (release 6.6). In cases where the data exhibited a non-normal distribution, standardization was performed using the formula (raw quantitation value of the sample)/[(sum of quantitation values of all metabolites in the sample)/(sum of quantitation values of all metabolites in the first QC (QC1) sample)], so as to derive relative peak areas. Compounds for which the coefficient of variation (CV) of relative peak areas in QC samples exceeded 30% were excluded. Ultimately, results pertaining to the identification and relative quantitation of metabolites were obtained. In addition, K-means-based correlation analysis was conducted to characterize metabolite change trends and their positive or negative correlations with milk components.

## 3. Results

### 3.1. Changes in Milk Composition at Different Lactation Stages

**Figure 1:**
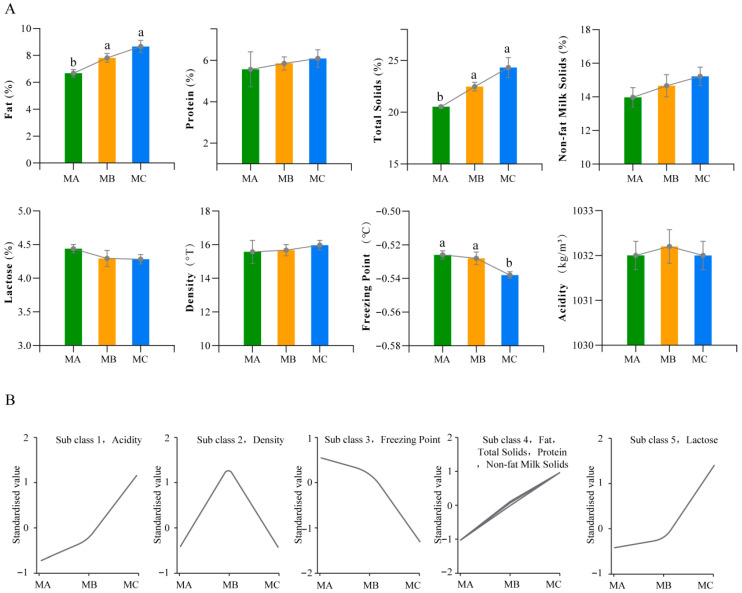
(**A**) Milk composition and physicochemical profiles of MA, MB, and MC (n = 5 per group). One-way ANOVA was performed on milk composition data. Levene’s test was used to assess variance homogeneity; the Shapiro–Wilk test was used to evaluate normality; Tukey’s test was used for post hoc comparisons when both were satisfied, while non-normal data were analyzed via bootstrap (1000 resamples) with BCa confidence intervals. Distinct lowercase letters (a, b) above bars mark significant inter-group differences (*p* < 0.05). (**B**) K-means clustering analysis.

**Figure 2:**
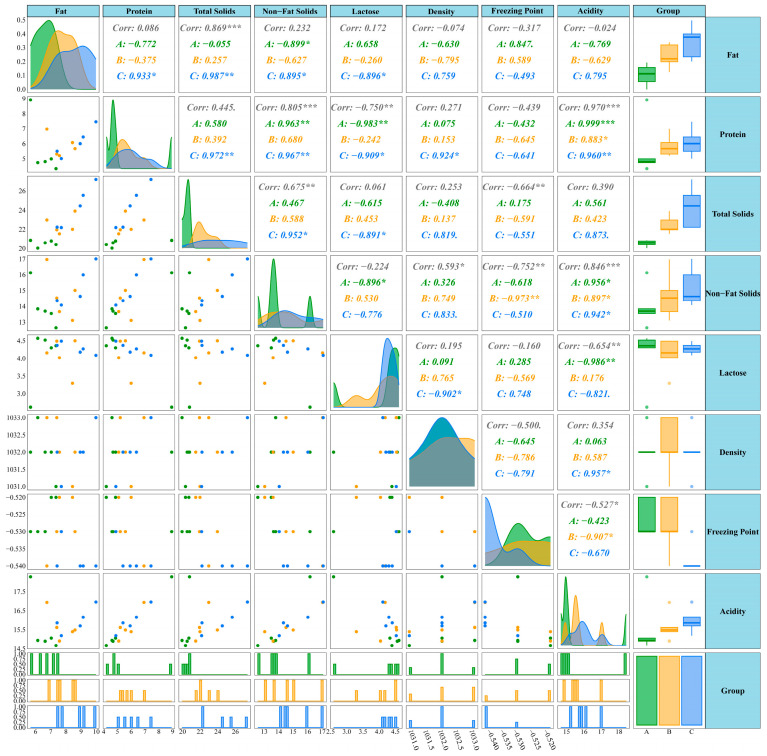
Correlation analysis of milk composition. Histograms (on the diagonal) display individual distributions, scatter plots (off the diagonal) present correlations between indicators, and box plots (at the margins) show grouped statistical data, with green representing the MA group, yellow representing the MB group, and blue representing the MC group, and correlation coefficients and significance levels for different groups (A/B/C) are annotated to facilitate the exploration of relationships and distribution patterns among dairy product components, where * indicates *p* < 0.05, ** indicates *p* < 0.01, and *** indicates *p* < 0.001.

Milk fat percentage, lactose percentage, milk protein, total solids, solids-not-fat, freezing point, density, and acidity were measured across three periods. As shown in Figure 1A, with changes in lactation time, milk fat percentage, total solids, and freezing point came significant changes, with milk fat percentage exhibiting an increasing trend over lactation time, where the MA had significantly lower milk fat percentage than the MB and MC, and total solids showed a similar trend. Meanwhile, freezing point showed a decreasing trend with the extension of lactation period, specifically manifested as the MC having a significantly lower freezing point than the MA and MB. Additionally, K-means clustering analysis of milk components revealed that milk components overall exhibited five change patterns, with milk fat percentage, total solids, milk protein, and solids-not-fat showing the same change trend (Figure 1B). Through complex correlation analysis evaluating the distribution patterns, correlations, and sample balance of milk component data, the results showed that milk fat percentage was significantly positively correlated with total solids; freezing point was negatively correlated with total solids, solids-not-fat, and acidity; and milk protein was positively correlated with acidity and solids-not-fat but negatively correlated with lactose (Figure 2).

### 3.2. PCA

**Figure 3:**
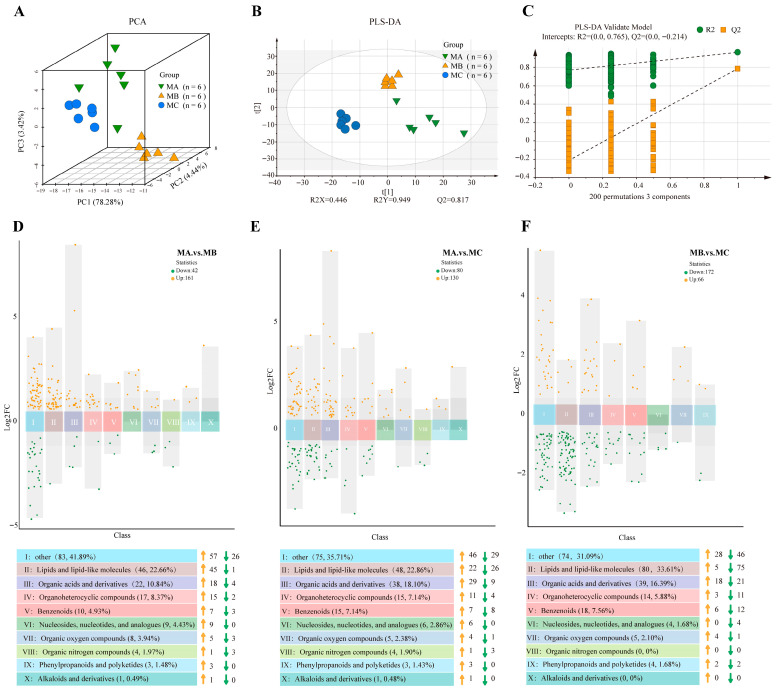
(**A**) PCA; (**B**) PLS-DA; (**C**) permutation test diagram; (**D**–**F**) grouped volcano plot of differential metabolites. The vertical axis represents the Log^2^FC values; the middle coordinate axis indicates different metabolite categories. The colored squares at the bottom represent the metabolite classifications corresponding to the middle coordinate axis with their quantities and proportions, and the orange and green arrows indicate the specific numbers of changes in different categories of differential metabolites.

Through unsupervised PCA, the distribution trends of three sample groups were observed (Figure 3A). The results showed that principal component 1 (PC1) = 78.28%, principal component 2 (PC2) = 4.44%, and principal component 3 (PC3) = 3.42%. In the scree plot, the variance proportions for PC1–PC5 are 0.78282, 0.04437, 0.03418, 0.02931, and 0.01707, respectively, yielding a cumulative proportion of 0.90775; PC1 contributes the most with a progressively decreasing trend thereafter, and the first five PCs explain more than 90% of the total variance (Figure S1, Table S1). From the sample point clustering patterns, samples within groups clustered together while showing clear separation between groups, indicating significant differences among the three lactation stage sheep samples in three-dimensional PCA, with significant differences in physiological states of sheep at different lactation stages, and PCA effectively distinguished sample characteristics of different lactation stages.

### 3.3. PLS-DA

PLS-DA further revealed the differences among samples across groups (Figure 3B). The results showed that the PLS-DA model had R2X = 0.446, R2Y = 0.949, and Q2 = 0.817. Here, R2X represents the model’s explanatory ability for X variables (metabolite data), meaning the model explains 44.6% of metabolite variation; R2Y reflects the goodness-of-fit for the class structure, indicating that 94.9% of the variation in Y is captured. Q2 represents the model’s predictive ability, indicating that the model has 81.7% prediction accuracy. Generally, Q2 > 0.5 indicates good model predictive ability, and in this study, Q2 > 0.9 indicates the model has excellent discriminative ability. Additionally, permutation test results indicated that the model was stable, with no overfitting, and intercepts of R2 = 0.765 and Q2 = −0.214 were found; the negative Q2 values in the permuted models support the reliability of the PLS-DA model, demonstrating that the model can effectively distinguish group-specific metabolic characteristics and that this differentiation is not caused by random factors. All of the above results indicated that the groups had significant differences in metabolic profiles which could be used for subsequent data analysis.

Based on the confusion matrix (Table S2), we calculated indicators including the class-specific sensitivity, specificity, balanced accuracy, and overall error rate. The results showed that for the three categories MA, MB, and MC, the class-specific sensitivity was 100%, indicating that all actual positive samples were correctly identified; the specificity was also 100%, with no instances of samples from other categories being misclassified as the target category. Further calculation of balanced accuracy revealed it also reached 100%; meanwhile, the number of misclassified samples was 0, and the overall error rate was 0%. As observed from the confusion matrix, all predictions fell on the diagonal positions, with off-diagonal elements being zero, indicating no misclassification across all categories by the model. This further validated the accuracy of the PLS-DA model.

### 3.4. Differential Metabolite Screening

After rigorous denoising, blank removal, missing value and QC variation screening, and the merging of isotopes/adduct ions/fragments, a total of 962 peaks were extracted from 18 samples in both positive- and negative-ion modes, with 466 peaks in positive-ion mode and 496 peaks in negative-ion mode. The typical total ion chromatograms (TICs) of all samples are shown in Supplementary Figure S2. Metabolites were screened using the selection criteria of VIP > 1, *p* < 0.05, FC ≥ 1.5, or FC ≤ 1/1.5. Through comparison between the MA and MB, 207 differential metabolites were identified, of which 161 were upregulated and 42 were downregulated. Comparison between the MA and MC yielded 210 differential metabolites, with 130 upregulated and 80 downregulated. Comparison between the MB and MC identified 238 differential metabolites, with 66 upregulated and 172 downregulated. The specific differences in metabolites are detailed in Supplementary Table S3. The overall changes in these differential metabolites and the specific classification of different metabolite categories were further demonstrated (Figure 3D–F).

### 3.5. Classification of Differential Metabolites

Classification (primary classification) analysis of differential metabolites from three lactation stages was performed based on the HMDB database. In the MA vs. MB comparison, differential metabolites were mainly enriched in lipids and lipid-like molecules (22.66%), organic acids and derivatives (10.84%), and organoheterocyclic compounds (8.37%) (Figure 3D). The MA vs. MC showed more balanced distribution of differential metabolites, with lipids and lipid-like molecules accounting for 22.86%, organic acids and derivatives for 18.10%, and organooxygen compounds for 7.14% (Figure 3E). The MB vs. MC exhibited unique metabolite classification characteristics, with lipids and lipid-like molecules accounting for 33.61%, organic acids and derivatives for 16.39%, and benzenoids for 7.56% (Figure 3F). All three comparisons showed a dominance of lipid metabolism-related molecules, indicating dynamic changes in lipid metabolism at different lactation stages. Additionally, organic acids, phenylpropanoids, and polyketides also played important roles in stage transitions, reflecting the adaptive regulation of energy metabolism and physiological state in Xinggao sheep during lactation.

### 3.6. KEGG Pathway Analysis of Differential Metabolites

**Figure 4:**
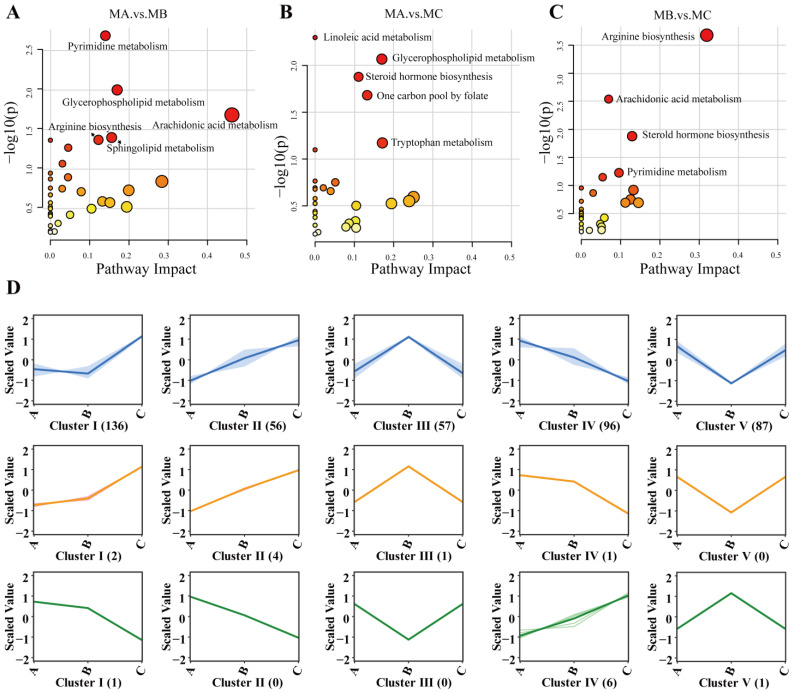
(**A**–**C**) KEGG enrichment analysis of differentially metabolized substances in different groups; (**D**) K-mean correlation analysis. Note: In Part (**D**), the blue panels represent metabolomics K-means clustering plots, with each panel representing metabolites exhibiting identical variation trends; the orange panels indicate K-means clustering of milk component expression levels that are positively correlated with the metabolome; and the green panels represent K-means clustering of milk component expression levels that are negatively correlated with the metabolome.

KEGG pathway enrichment analysis revealed the metabolic regulatory characteristics of three lactation stages. In the MA vs. MB comparison, significantly enriched pathways included pyrimidine metabolism, glycerophospholipid metabolism, and arachidonic acid metabolism (Figure 4A, Supplementary Table S4). Pathway enrichment of differential metabolites in MA vs. MC was primarily concentrated in linoleic acid metabolism, glycerophospholipid metabolism, and steroid hormone biosynthesis, indicating important transitions in hormonal regulation and lipid metabolism from early to late lactation (Figure 4B, Supplementary Table S4). The MB vs. MC was predominantly enriched in the arginine biosynthesis, arachidonic acid metabolism, steroid hormone biosynthesis, pyrimidine metabolism, and tyrosine metabolism (Figure 4C, Supplementary Table S4), reflecting the regulatory roles of reproductive hormones and inflammation-related metabolism during the transition from mid- to late lactation. All three comparisons demonstrated the importance of the amino acid regulation pathway, the steroid metabolism pathway, and the lipid metabolism pathway, suggesting that these pathways play crucial roles in physiological regulation during lactation in Xinggao sheep.

### 3.7. K-Means Clustering Analysis of Differential Metabolites and Milk Components

The K-means clustering algorithm was employed to classify differential metabolites into nine clusters based on their expression patterns across three lactation stages. The blue panels represent metabolomics K-means clustering plots, with each panel representing metabolites exhibiting identical variation trends; the orange panels indicate K-means clustering of milk component expression levels that are positively correlated with the metabolome; and the green panels represent K-means clustering of milk component expression levels that are negatively correlated with the metabolome. Three significantly altered milk components were primarily distributed in clusters I, II, and IV, with freezing point showing a positive correlation with cluster IV and a negative correlation with cluster I. Milk fat percentage showed a positive correlation with cluster II and a negative correlation with cluster IV. The total solids content exhibited a positive correlation with cluster II and a negative correlation with cluster IV (Supplementary Table S5).

Cluster I primarily contained 136 metabolites, including metabolites such as aldosterone, guanosine, and betaine. Cluster II primarily contained 56 metabolites, including metabolites such as progesterone, L-aspartic acid, and methyl palmitate. Cluster IV primarily contained 96 metabolites, including metabolites such as citrulline, ornithine, and homoarginine.

### 3.8. Random Forest Analysis of Differential Metabolites

**Figure 5:**
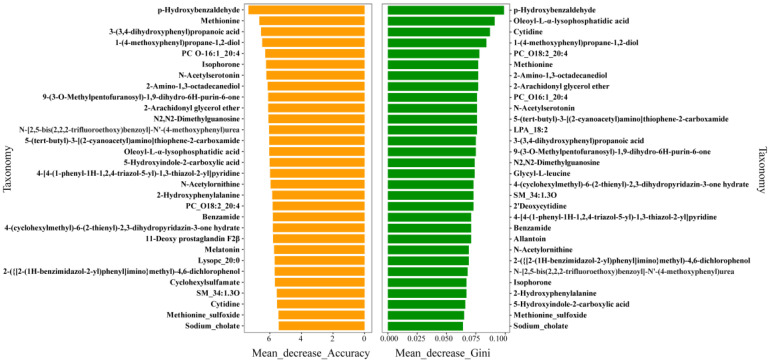
Random forest mean decrease accuracy and mean decrease Gini index.

This study employed a random forest algorithm to analyze blood and milk sample indicators from different Xinggao sheep lactation stages, evaluating the importance of feature variables through mean decrease accuracy and mean decrease Gini index (Figure 5). These two metrics measure variable importance from different perspectives—mean decrease accuracy reflects the contribution of a variable to model prediction accuracy, while mean decrease Gini index measures each variable’s impact on the heterogeneity of observations at each node of the classification tree, thereby comparing variable importance. The results indicated that the importance rankings of various substances differed between the two metrics, which may be attributed to their different methods of evaluating importance. Among them, 4-hydroxybenzaldehyde showed the highest values in both mean decrease accuracy and mean decrease Gini index (Mean_decrease_accuracy = 7.352; Mean_decrease_gini = 0.099), indicating its significant role in classification. Multiple substances demonstrated high importance in both metrics, including 2-amino-13-octadecanediol, N-acetylserotonin, 2-arachidonylglycerol ether, and PC O16:1_20:4, indicating their crucial roles in classifying Xinggao sheep lactation stages. Additionally, stress-related markers methionine sulfoxide and methionine also exhibited high importance in both metrics. These results provide potential biomarkers for an in-depth understanding of the physiological lactation mechanisms of Xinggao sheep.

## 4. Discussion

### 4.1. Changes in Milk Composition During Different Lactation Periods

Milk fat, as the most important and energy-rich component in milk, plays a crucial role in the growth, development, and nutritional metabolism of lambs. Its content is regulated by multiple factors, including physiological status, lactation stage, and nutritional level. This study found that milk fat percentage showed a significant increasing trend with the extension of lactation period, peaking during the MC period. We hypothesize that this change is closely related to the transition in lamb feeding behavior: as age increases, lambs gradually increase their starter feed intake while correspondingly reducing their dependence on maternal milk, leading to decreased suckling frequency and intensity. As shown by Zipp et al. [14], milk fat percentage shows a negative correlation with calf suckling behavior, with cows that have no contact with calves exhibiting higher milk fat content compared to those with calf access. Furthermore, other studies have also found that milk fat content in non-nursing dairy cows is higher than that during the nursing period [15]. Ontsouka et al. [16] provided an explanation for this phenomenon, stating that due to the lower specific gravity of fat, milk fat accumulates substantially in mammary alveoli, and frequent suckling helps expel milk fat from the alveoli, thus reduced suckling behavior may decrease fat content in milk. We hypothesize that the increase in milk fat percentage observed in this study is primarily attributed to the gradual decrease in lambs’ dependence on maternal milk during late lactation and the corresponding reduction in their suckling behavior [15,16], and further testing is needed to confirm this hypothesis.

Total solids content is also one of the key indicators for evaluating the overall quality of sheep milk, reflecting the total levels of components such as lactose, protein, fat, minerals, and vitamins in sheep milk. The extension of the lactation period is considered an important factor affecting the total solids in sheep milk. As found by Ante et al. [2], with the progression of lactation, the total solids, fat, and casein content in sheep milk were significantly higher, which is closely related to component accumulation and mammary gland physiological changes during lactation. In studies of D’Man ewes, it was also found that both total solids and fat content showed an increasing trend with the extension of the lactation period [17]. Yilmazi et al. [18] also reported similar research findings. These research results are consistent with our findings, showing that total solids present an upward trend with the extension of the lactation period and are positively correlated with milk fat percentage. It is worth noting that the dynamic changes in total solids reflect the complex interactions of sheep milk components. These changes involve not only adjustments in macronutrient content (such as protein, fat, and lactose) but also fluctuations in trace elements and minerals. Therefore, the upward trend in total solids is not only closely linked to the aforementioned increase in milk fat (as the significant rise in milk fat proportion drives the overall growth of total solids levels), but may also be driven by dynamic adjustments in the internal nutritional components of sheep milk, including changes in protein, lactose, and other elements.

Freezing point is an important indicator for evaluating water content in milk components, with its variation depending on the number of solute particles in the solvent; under normal conditions, the concentration of soluble low-molecular components in milk (such as lactose, potassium, and chloride) remains stable, maintaining the freezing point within a narrow range; when fluid balance fluctuates, such as when the solute concentration increases, the freezing point significantly decreases [19]; thus, changes in freezing point directly reflect the dynamic balance between solute components and water content in milk. This study found that the freezing point became significantly lower with the extension of the lactation period and showed a negative correlation with changes in total solids. Similar findings were also observed in goat studies, where in the late lactation stages, the accumulation of solids in milk, along with changes in mammary epithelial and blood–milk barrier permeability, further lowered the freezing point [20]. This indicates that lactation stage indirectly affects the balance between water and solutes by altering the concentration of total solids in milk (such as lactose, minerals, etc.), with the increased solute particles represented by total solids further decreasing the freezing point.

### 4.2. Metabolic Changes in Lactating Ewes

In recent years, metabolomics research on lactation in ruminants has achieved significant breakthroughs through multidimensional sample analysis, multi-omics integration, and functional mechanism exploration, providing novel perspectives for elucidating metabolic adaptation patterns during lactation and production performance regulatory mechanisms. In dairy cattle research, previous serum metabolomics and lipidomics analyses have revealed that differences between high- and low-fat-mobilization cows in the early postpartum period were concentrated in sphingolipid metabolism, with ceramide levels (such as Cer (d18:1/16:0)) positively correlated with insulin resistance, hepatic dysfunction, and inflammation, while elevated acylcarnitines indicated enhanced fatty acid β-oxidation and energy metabolism imbalance [21]. Goat lactation metabolomics research primarily focuses on the associations between key pathways and lactation performance. Wang et al. found that Streptococcus in young goat rumen could regulate lactation function through amino acid metabolic pathways, with its abundance achieving a prediction accuracy of 91.7% for adult milk yield, while Prevotella inhibited rumen fermentation and reduced milk protein content [22]. Sha et al. compared Hu sheep and Suffolk sheep and found that Suffolk sheep showed active reproductive hormone levels and energy metabolism pathways during pregnancy and lactation, with increased Prevotella abundance positively correlated with lamb birth weight; Hu sheep exhibited significantly enhanced amino acid metabolic pathways such as arginine-related metabolism during pregnancy, providing energy support for multiple fetal development [23]. Studies on Small-tailed Han sheep indicated that the rumen microbiota and metabolites across reproductive stages were associated with immune function and metabolic adaptations, with serum and rumen metabolites synergistically regulating immune and lactation performance [24]. The present study’s results showed that serum differential metabolites in Xinggao sheep across different lactation stages were mainly enriched in steroid hormone synthesis and arginine synthesis pathways, with the citrulline–arginine axis showing a strong correlation with milk fat percentage. Therefore, in further breeding research, priority should be given to screening superior individuals with active steroid hormone and arginine metabolism and utilizing pathway markers such as methionine to further improve breeding precision; regarding nutritional management strategies, research should focus on different precision approaches during lactation stages, emphasizing rumen-protected amino acid supplementation in the early stage and increasing arginine precursor supply in the later stage, thereby targeting activation of core metabolic pathways to ultimately achieve synergistic optimization of milk quality and lactation performance.

The PCA and PLS-DA results showed that the MA exhibited higher sample dispersion compared to the MB and MC, which may reflect the complex metabolic transition processes in ewes during early lactation. After parturition, mammals typically need to rapidly adapt to the metabolic transition from pregnancy to lactation, during which this process involves rapid mammary gland development and the initiation of milk synthesis, leading to dramatically increased demands for glucose, fatty acids, and amino acids [23,25]. The body responds to this demand by enhancing hepatic gluconeogenesis and adipose tissue mobilization. Meanwhile, the parturition process itself is accompanied by significant physiological stress responses, including elevated oxidative stress levels and dramatic hormonal changes, which further exacerbate metabolic instability in the organism [26,27]. Different individuals exhibit significant variations in prepartum body condition, body fat reserves, parturition stress responses, and mammary gland development, and these differences directly affect the efficiency and speed of individual metabolic adaptation. Therefore, the greater dispersion of samples in the MA may be a direct manifestation of these inter-individual differences in metabolic adaptation capacity.

#### 4.2.1. Metabolic Differences Between MA and MB

Pyrimidine metabolism plays a crucial role in the function and metabolism of mammary cells during the lactation cycle, involving multiple aspects such as nucleotide synthesis, cell proliferation, and differentiation [28,29,30]. This study found that, compared to the MA, the levels of pyrimidine metabolism-related metabolites (such as uridine, with an effect size of −2.409; cytosine, with an effect size of −2.104; and thymine, with an effect size of −1.481) in ewe serum were significantly lower in the MB. This finding is consistent with the results of Li et al.’s research [31] on yak mammary tissue, where they observed a gradual decrease in uridine levels in mammary tissue with the extension of lactation period. This alteration in metabolic pattern is closely associated with the dynamic differences in hormone levels during the lactation cycle, particularly the action of prolactin. It is generally recognized that high levels of prolactin during pregnancy and early lactation can promote mammary gland enlargement and prepare for lactation. Furthermore, prolactin regulates uridine metabolism while stimulating uridine uptake in the mammary gland and its synthesis into RNA [32]. Therefore, we propose that the decrease in pyrimidine metabolite levels may reflect the conventional transition in mammary cell proliferation and differentiation status from early to mid-lactation, as well as normal fluctuations in hormone levels, and additional experiments are required to validate this hypothesis.

Arachidonic acid plays an important role in embryonic and fetal development, as well as metabolic and physiological processes in early life, and it is particularly crucial for infant early growth, brain development, and health [33]. Our study found that, with the extension of lactation time, ARA (with an effect size of −1.122) levels in ewe serum decrease significantly. This is similar to the results of Francesca et al.’s [34] human studies, wherein it was found that ARA is preferentially transferred from mother to infant, manifested as significantly lower maternal serum ARA levels with higher breastfeeding time, while infant plasma ARA levels were significantly higher than maternal levels, which may have important implications for infant development. Similar research findings have also been found in cattle [33]. This also coincides with our finding of increased lipid substances in milk components.

#### 4.2.2. Metabolic Differences Between MA and MC

Steroid hormones, such as sex hormones, mineralocorticoids, and glucocorticoids, are cholesterol derivatives mainly synthesized in the gonads, placenta, and adrenal cortex [35], playing an important role in adaptive regulation during lactation [36]. This study revealed that, compared with the MA, the MC showed significantly elevated levels of dehydroepiandrosterone (DHEA) (effect size = 1.667), Etiocholanolone (effect size = 2.361), Androsterone (effect size = 1.758), and pregnenolone (effect size = 1.081) in the steroid hormone biosynthesis pathway, while cortisol (effect size = −1.492) and Tetrahydrocorticosterone (effect size = −1.295) levels were significantly lower. Fluctuations in cortisol levels can effectively reflect the stress level in ruminants and are key biological indicators for stress assessment [37]. DHEA is a precursor of androgens and has been proven to have anti-inflammatory and antioxidant effects [38]. Human studies have found that acute psychosocial stress leads to significant increases in DHEA levels, which act as antagonists to the stress hormone cortisol, protecting the body during stress [39]. This finding has also been confirmed in dairy cows, where an increase in DHEA was observed approximately 5 days after a significant elevation in plasma cortisol [40]. Therefore, we propose that the lower cortisol levels and higher DHEA levels in the MC reflect the physiological adaptation process of ruminants gradually recovering from the stress states of parturition and NEB (negative energy balance) in early lactation, which requires further investigation to confirm.

Pregnenolone is a substrate for steroid hormone synthesis that can further generate progesterone, cortisol, and sex hormones, and its level differences may reflect adaptive adjustments of the hypothalamic–pituitary–adrenal axis (HPA axis) [41]. Similarly to DHEA’s function, pregnenolone has been proven to directly attenuate stress-induced physiological and behavioral responses. For example, studies have found that exogenous pregnenolone treatment can significantly reduce stress-induced craving and anxiety [42]. Other studies have also found that pregnenolone can reduce stress by restoring the HPA axis [43]. Therefore, the significant elevation of DHEA and pregnenolone levels in the MC may be a compensatory response to stress events in early lactation, maintaining hormonal balance and preventing potential damage from prolonged high cortisol levels by regulating and alleviating excessive HPA axis activation.

#### 4.2.3. Metabolic Differences Between MB and MC

Estrogens are a class of steroid hormones containing 18 carbon atoms, derived from cholesterol and mainly secreted by the ovaries and placenta, among which estradiol has been widely studied due to its high activity and affinity [44]. During lactation, however, it has an inhibitory effect on milk secretion, which has been confirmed in cattle studies showing that high circulating estradiol levels can reduce milk production [45,46]. Yart et al. [47] also confirmed that estradiol can accelerate the apoptotic process of bovine mammary epithelial cells (bMECs), particularly targeting early apoptotic cells. Estradiol promotes cell death by activating the caspase pathway, and this effect is more pronounced in senescent cells. Human studies have also found that circulating estradiol levels at 4 weeks postpartum are negatively correlated with milk production [48]. Therefore, elevated estradiol levels are typically associated with mammary tissue involution and decreased milk production. This study found that, compared to the MB, the MC showed significantly increased levels of estradiol (with an effect size of 2.834), testosterone (with an effect size of 4.411), dehydroepiandrosterone (with an effect size of 3.424), androsterone (with an effect size of 8.301), 7α-hydroxytestosterone (with an effect size of 1.38), etiocholanolone (with an effect size of 5.871), and aldosterone (with an effect size of 3.94) in the Steroid hormone biosynthesis pathway. Therefore, we hypothesize that as lamb supplementary feeding increases (with lambs’ dependence on milk gradually decreasing in late lactation and the lactation load consequently reducing), combined with the sustained regulation of mammary function by the high lactation load during mid-lactation and the adaptive response of lactating ewes to early artificial weaning, related hormone levels are altered in late lactation, and further testing is needed to confirm this hypothesis.

### 4.3. Correlation Between Milk Composition and Serum Metabolome

As an important semi-essential amino acid, arginine plays a crucial role in physiological regulation and immune function maintenance during the lactation period of ewes [49]. This study found that compared with the MB, ewes in the MC showed significantly elevated arginine (with an effect size of 1.828) levels and significantly lower citrulline (with an effect size of −1.835) levels in the arginine biosynthesis pathway, and the variation trend of citrulline throughout the lactation period was negatively correlated with milk fat percentage. Research demonstrates that citrulline can not only synthesize arginine through normal renal pathways, but in lactating ewes (sheep and goats), mammary tissue can also convert some citrulline to arginine, thereby achieving efficient utilization of arginine by mammary tissue [50]. As the sole endogenous precursor of nitric oxide (NO), arginine can increase mammary blood flow by promoting vasodilation and angiogenesis, thereby enhancing the nutrient uptake capacity of mammary tissue [51]. Studies in mice show that elevated arginine levels in the circulatory system can promote the synthesis and utilization of milk fat [52]. Ding et al. [53] also found that elevated circulating arginine levels can promote milk fat production in dairy cows through two pathways—(1) by enhancing the expression of genes related to milk fat synthesis and (2) by increasing serum NO concentration (thereby increasing mammary blood flow and nutrient uptake by mammary tissue). Therefore, we hypothesize that the mechanism underlying the negative correlation between citrulline and milk fat percentage in K-means analysis may be as follows: citrulline, as a precursor of arginine, is extensively converted and utilized, leading to significantly lower citrulline levels and significantly higher arginine levels; subsequently, the citrulline–arginine metabolic axis promotes NO production, and NO further regulates the expression of lipogenesis-related genes and key enzyme activities in mammary epithelial cells, ultimately affecting changes in milk fat percentage. However, further testing is needed to confirm this hypothesis.

Additionally, we found that the negative correlation between the steroid pathway and freezing point changes in milk components may be related to elevated aldosterone levels in the MC compared to MA and MB in the steroid pathway. Aldosterone is an important substance regulating water and salt metabolism in the body. It enhances water reabsorption by reducing sodium excretion and plays an important regulatory role in systemic electrolyte and water balance. The decrease in freezing point reflects an increase in solute concentration in milk. Therefore, we propose that elevated aldosterone levels lead to sodium retention in sheep, further increasing sodium ion concentration in milk, which increases milk solutes and further lowers the freezing point. Additional experiments are required to validate this hypothesis.

## 5. Conclusions

This study investigated the changes in the milk composition of ewes during different lactation periods and their association with body metabolism through the milk composition and serum metabolomics analysis of sheep. Milk composition analysis revealed that milk fat percentage and total solids increase significantly after early lactation, while the freezing point decreases significantly during late lactation. Furthermore, metabolomics analysis found that significantly different metabolites in late lactation were mainly enriched in steroid hormone biosynthesis, and arginine biosynthesis pathways, indicating that, during late lactation, adaptive responses through changes in steroid hormone and amino acid levels maintain body homeostasis. Correlation analysis using K-means clustering revealed a positive correlation between milk fat percentage and total solids with steroid pathways regulating lipid metabolism, suggesting that steroid metabolism may be closely related to changes in sheep milk composition. The results of this study provide new insights for understanding the metabolic regulatory mechanisms of Xinggao sheep during different lactation stages, which is of great significance for the precision management of sheep. Future research should expand sample sizes to validate these findings and integrate multi-omics approaches (transcriptomics, proteomics) to construct comprehensive metabolite–milk composition regulatory networks. Additionally, employing in vitro mammary epithelial cell cultures and in vivo gene knockout/overexpression experiments will help elucidate how key metabolites (citrulline, estradiol) regulate milk fat synthesis and freezing point dynamics. Ultimately, developing and testing stage-specific nutritional interventions could enhance milk quality and yield in Xinggao sheep production systems.

## Data Availability

The original contributions presented in this study are included in the article/Supplementary Materials. Further inquiries can be directed to the corresponding author.

## Supplementary Materials

The following supporting information can be downloaded at: https://www.mdpi.com/article/10.3390/metabo15100678/s1, Table S1: Table showing principal component scree plot metrics. Table S2: Confusion matrix table. Table S3: Differential metabolites identified through inter-group comparisons. Table S4: Differential metabolite pathway analysis. Table S5: K-means correlation analysis of the relationship between metabolites and milk components. Figure S1: Scree plot. Figure S2: Quality control and sample total ion chromatogram (TIC).

## Abbreviations

The following abbreviations are used in this manuscript:

MA	Early lactation period
MB	Mid-lactation period
MC	Late lactation period
DHEA	Dehydroepiandrosterone
