# Supplementary material for: Research on the Changing Characteristics of Milk Composition and Serum Metabolites Across Various Lactation Periods in Xinggao Sheep
Source: Metabolites. 2025 Oct 20;15(10):678. doi: 10.3390/metabo15100678 (PMC12566154; doi:10.3390/metabo15100678)
Supplement: Supplementary file 1 [file metabolites-15-00678-s001.zip › Supplementary Material/FIG/S Fig2(NEG).pdf]

Specialized Traces
04-Jan-2024 9:40

Checked **Spectrum File**

HFX1_2139130_CN_QC2.raw

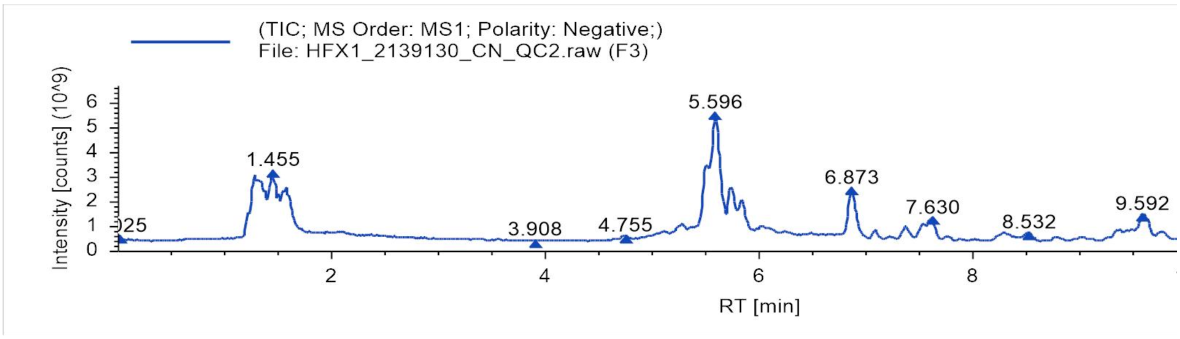

HFX1_2139130_CN_QC3.raw

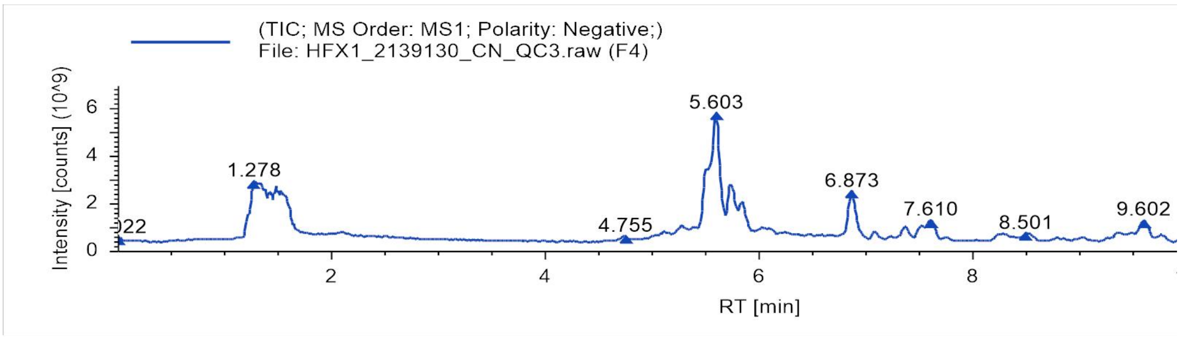

HFX1_CN1_FZTM230144882-1A.ra

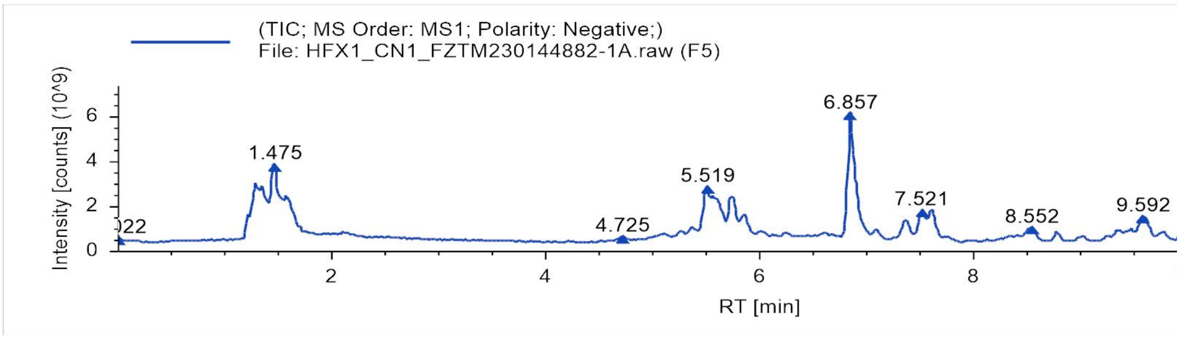

HFX1_CN1_FZTM230144883-1A.ra

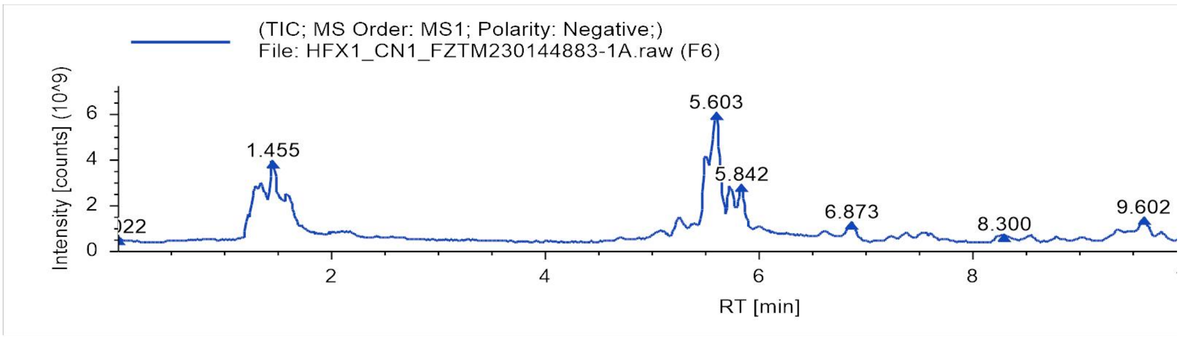

HFX1_CN1_FZTM230144884-1A.ra

© Reported with Compound Discoverer 3.3

Specialized Traces
04-Jan-2024 9:40

Checked **Spectrum File**

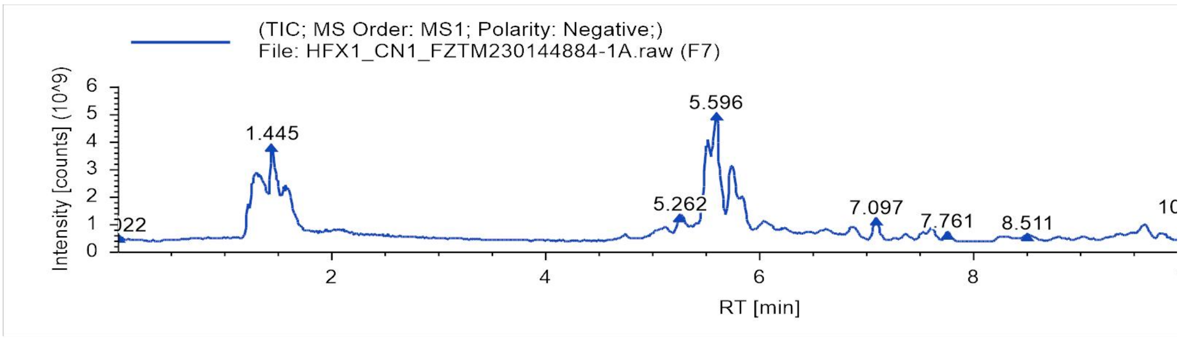

HFX1_CN1_FZTM230144885-1A.ra

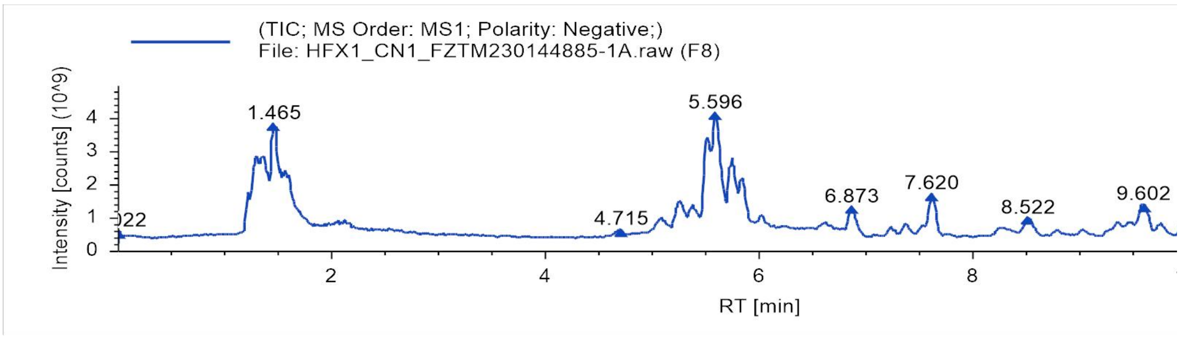

HFX1_CN1_FZTM230144886-1A.ra

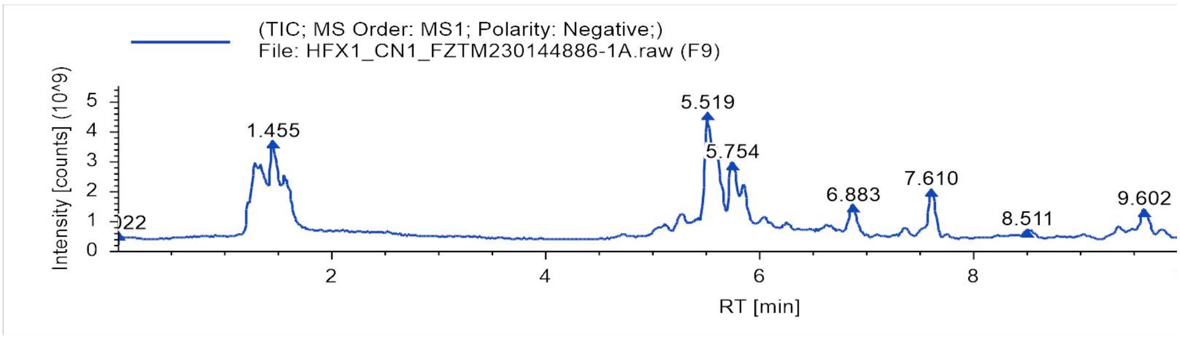

HFX1_CN1_FZTM230144887-1A.ra

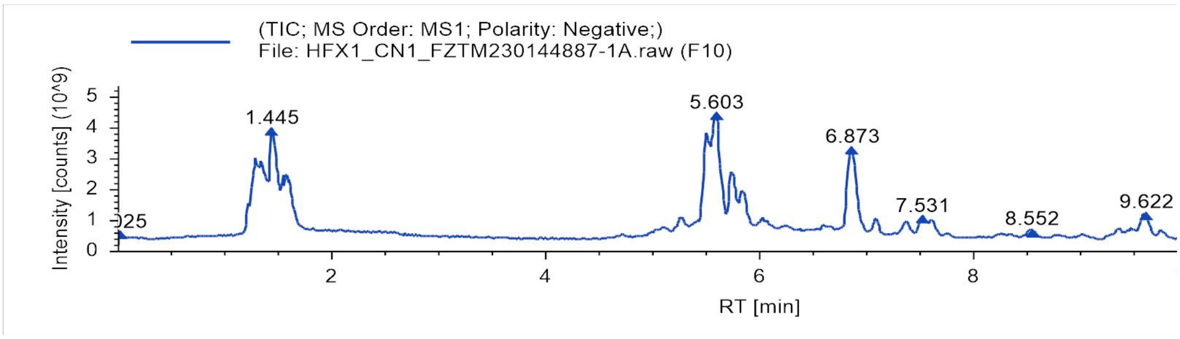

HFX1_CN2_FZTM230144888-1A.ra

© Reported with Compound Discoverer 3.3

Specialized Traces
04-Jan-2024 9:40

Checked **Spectrum File**

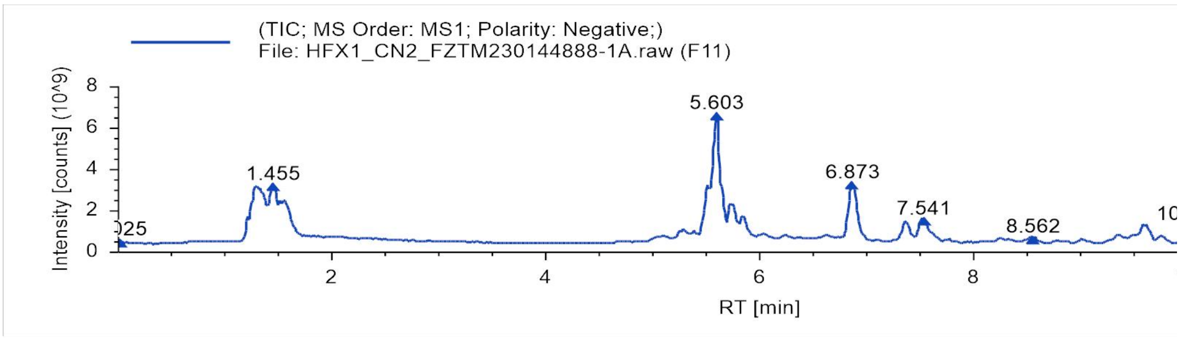

HFX1_CN2_FZTM230144889-1A.ra

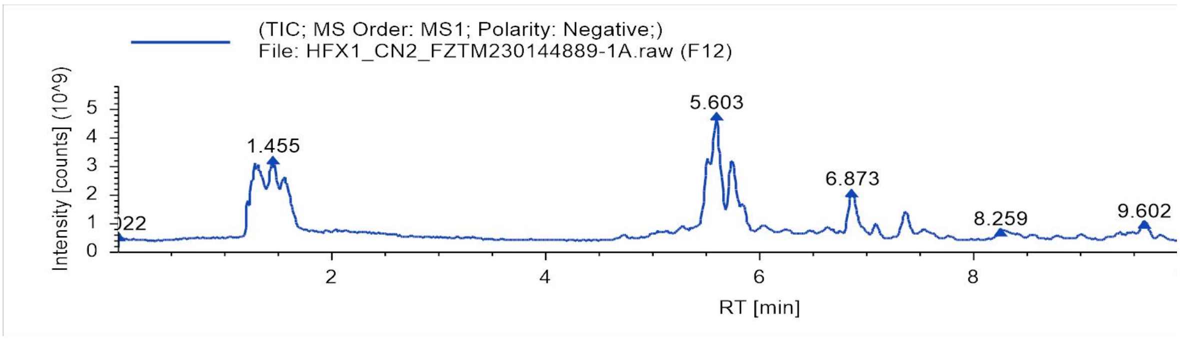

HFX1_CN2_FZTM230144890-1A.ra

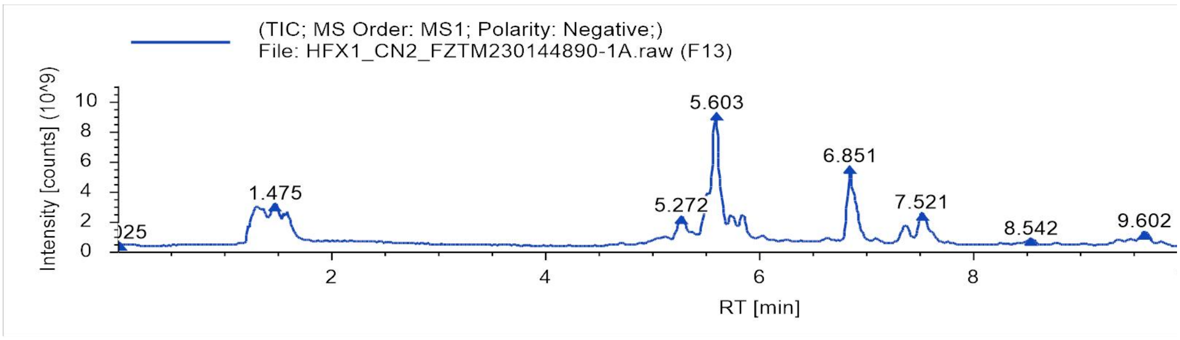

HFX1_CN2_FZTM230144891-1A.ra

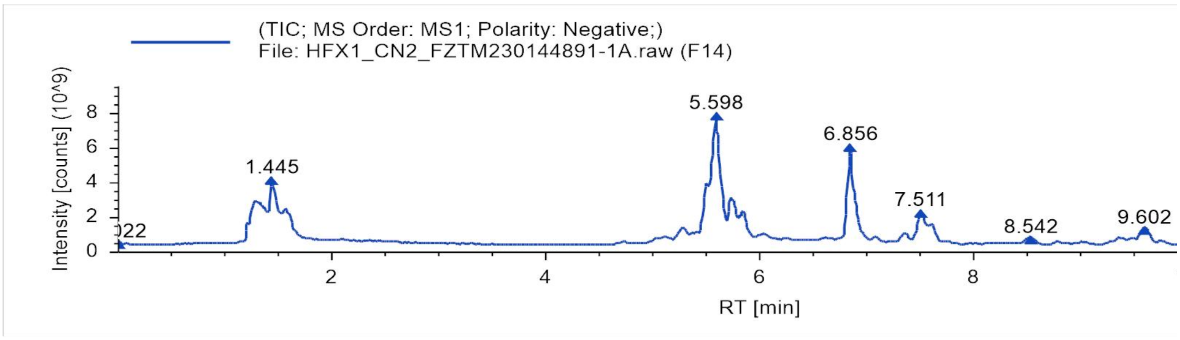

HFX1_CN2_FZTM230144892-1A.ra

© Reported with Compound Discoverer 3.3

Specialized Traces
04-Jan-2024 9:40

Checked **Spectrum File**

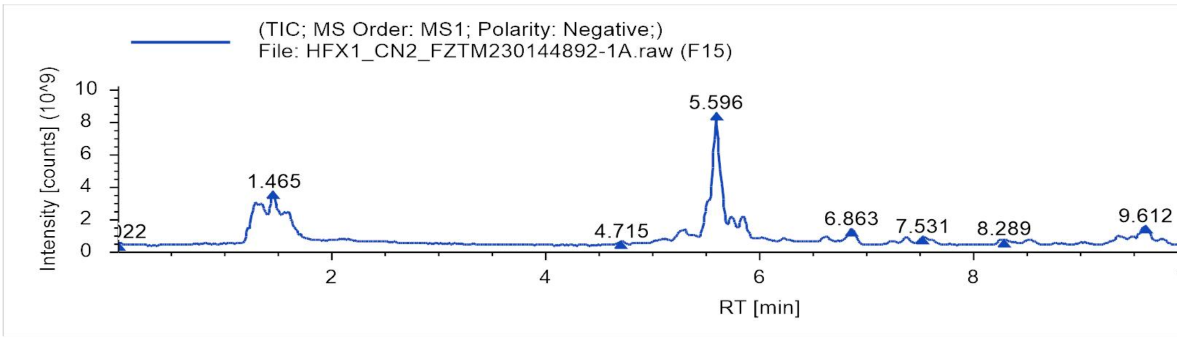

HFX1_CN2_FZTM230144893-1A.ra

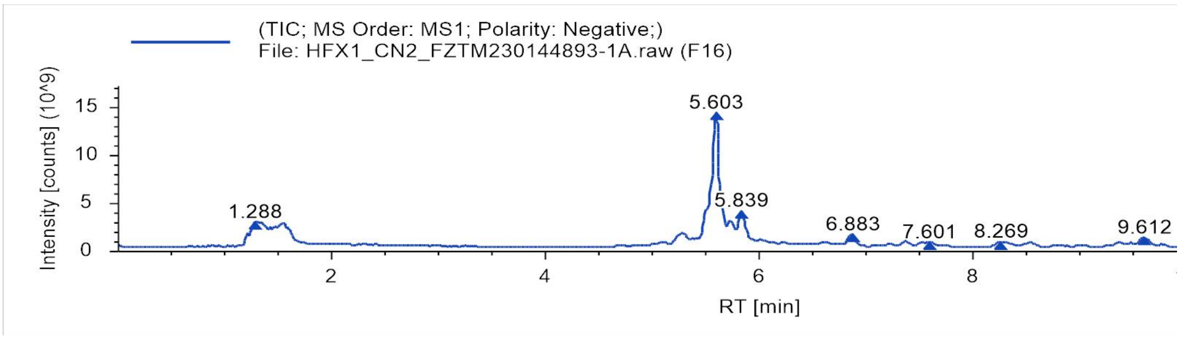

HFX1_CN3_FZTM230144894-1A.ra

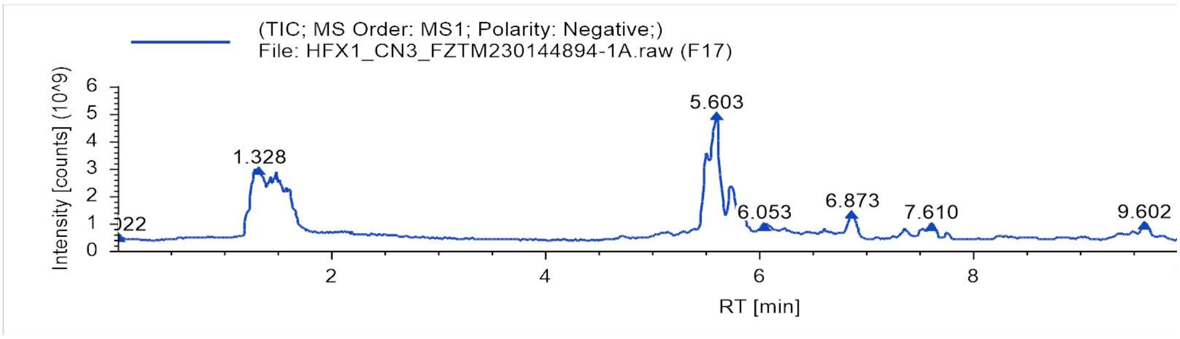

HFX1_CN3_FZTM230144895-1A.ra

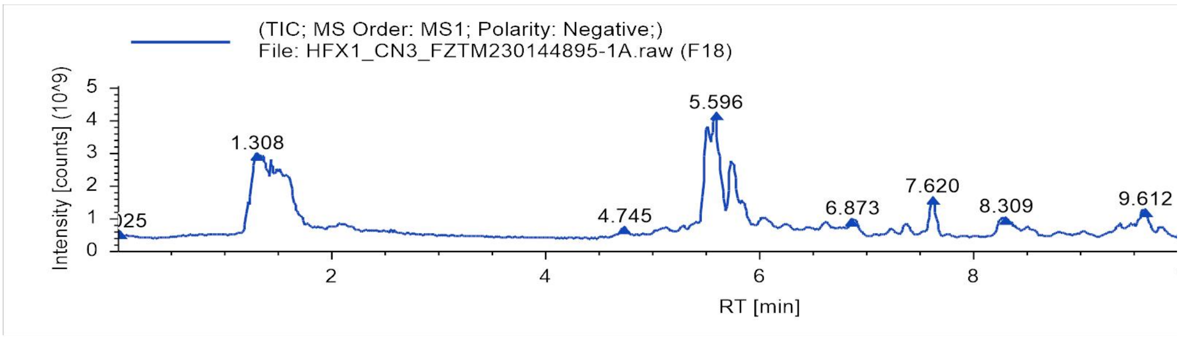

HFX1_CN3_FZTM230144896-1A.ra

© Reported with Compound Discoverer 3.3

Specialized Traces
04-Jan-2024 9:40

Checked **Spectrum File**

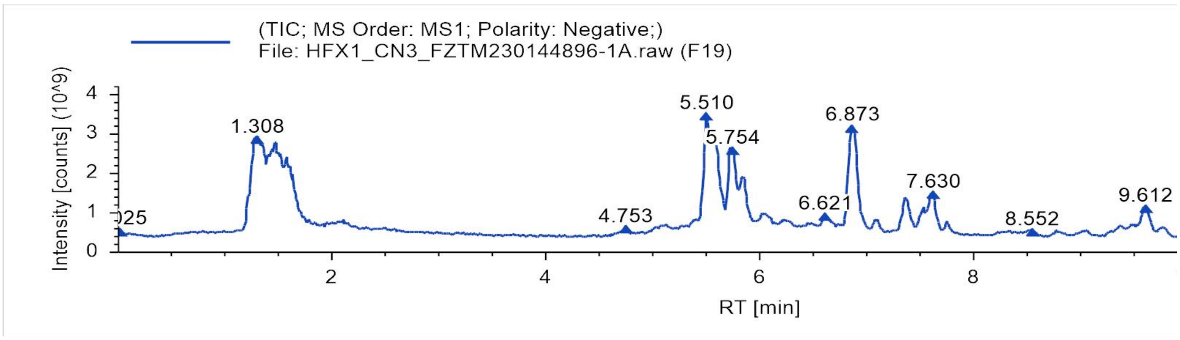

HFX1_CN3_FZTM230144897-1A.ra

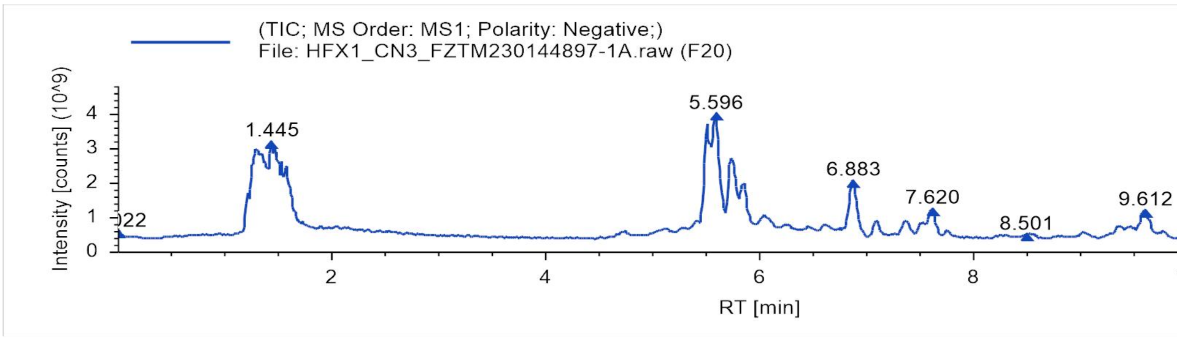

HFX1_CN3_FZTM230144898-1A.ra

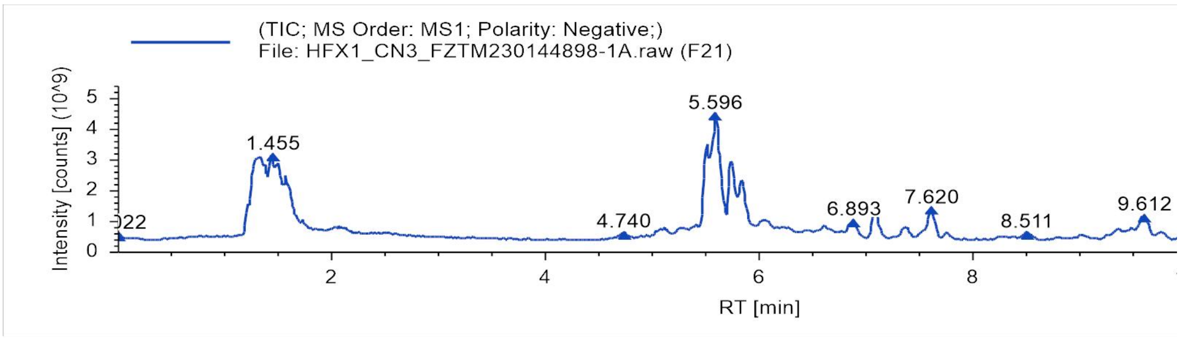

HFX1_CN3_FZTM230144899-1A.ra

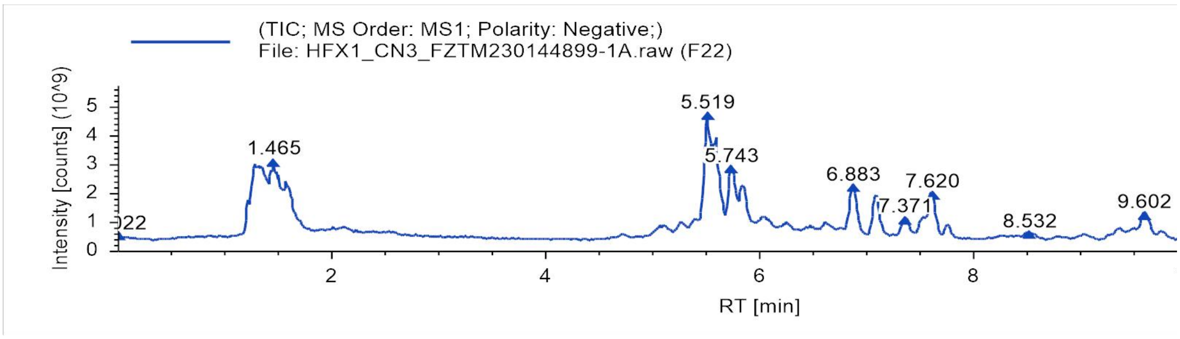

HFX1_2139130_CN_blank.raw

© Reported with Compound Discoverer 3.3

Specialized Traces
04-Jan-2024 9:40

Checked **Spectrum File**

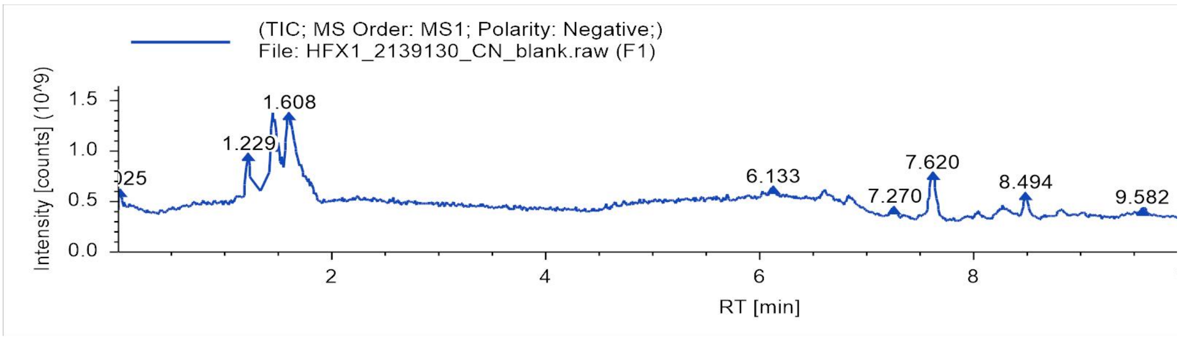

HFX1_2139130_CN_QC1.raw

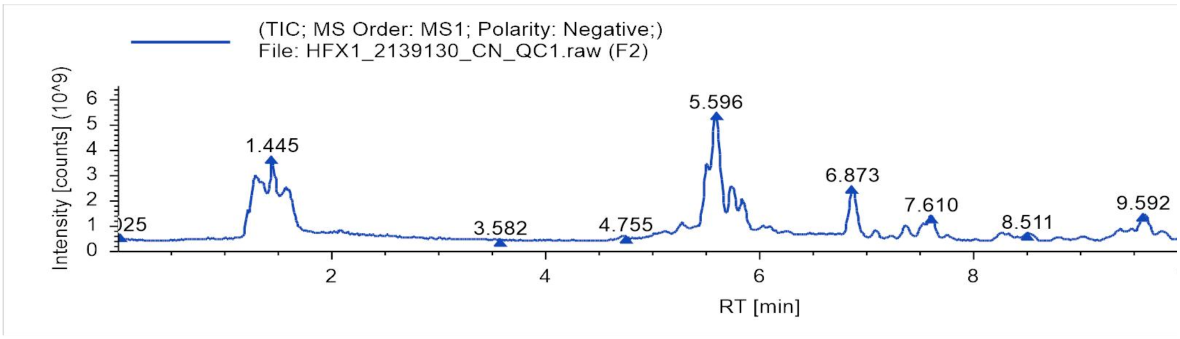

© Reported with Compound Discoverer 3.3

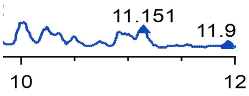
11.151
11.9
10
12

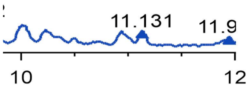
11.131
11.9
10
12

aW

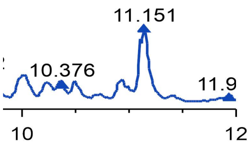
11.151
10.376
11.9
10
12

aW

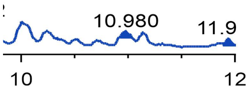
10.980
11.9
10
12

aW

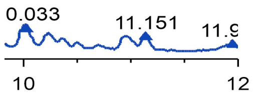
0.033
11.151
11.9
10
12

aW

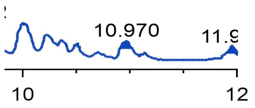
10.970
11.9
10
12

aW

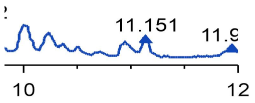
11.151
11.9
10
12

aW

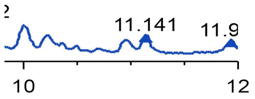
11.141
11.9
10
12

aW

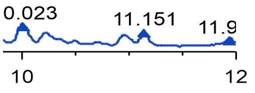
0.023
11.151
11.9
10
12

aW

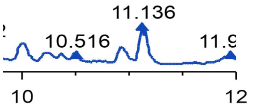
11.136
10.516
11.9
10
12

aW

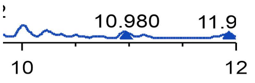
10.980
11.9
10
12

aW

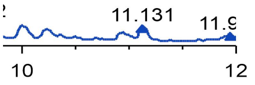
11.131
11.9
10
12

aW

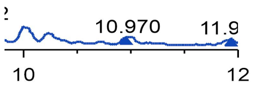

aW

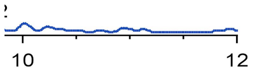

aW

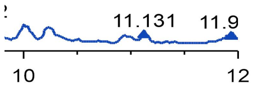

aW

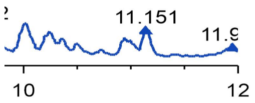

aW

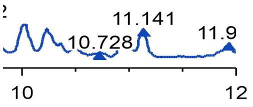
11.141
10.728
11.9
10
12

aW

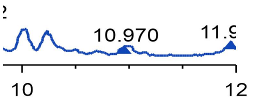
10.970
11.9
10
12

aW

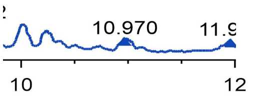
10.970
11.9
10
12

aW

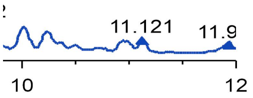
11.121
11.9
10
12

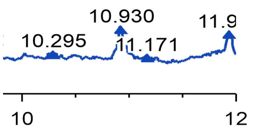
10.930
10.295
11.171
11.9
10
12

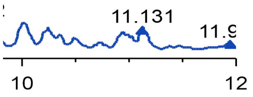
11.131
10
12
