# Supplementary material for: Research on the Changing Characteristics of Milk Composition and Serum Metabolites Across Various Lactation Periods in Xinggao Sheep
Source: Metabolites. 2025 Oct 20;15(10):678. doi: 10.3390/metabo15100678 (PMC12566154; doi:10.3390/metabo15100678)
Supplement: Supplementary file 1 [file metabolites-15-00678-s001.zip › Supplementary Material/FIG/S Fig2(POS).pdf]

Specialized Traces
02-Jan-2024 11:35

Checked **Spectrum File**

HFX1_2139130_CP_QC2.raw

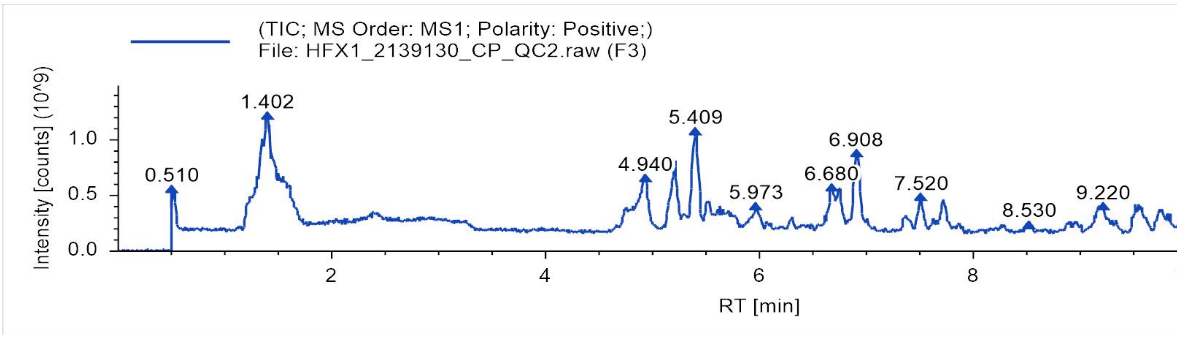

HFX1_2139130_CP_QC3.raw

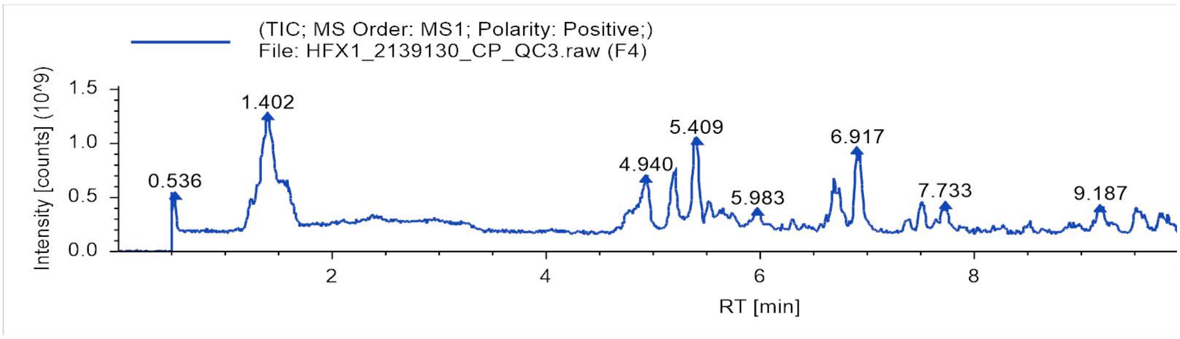

HFX1_CP1_FZTM230144882-1A.ra

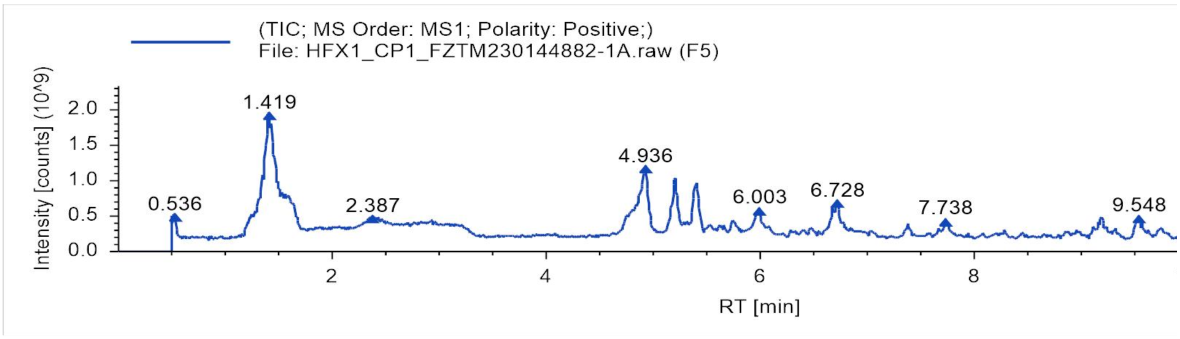

HFX1_CP1_FZTM230144883-1A.ra

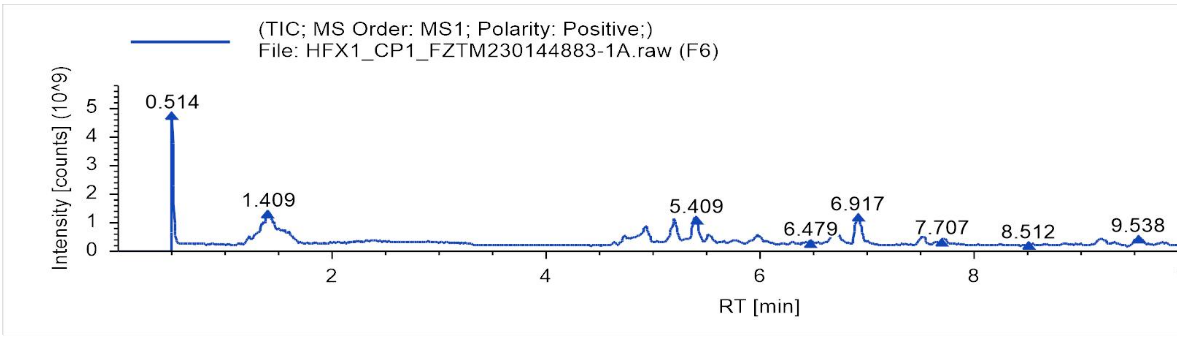

HFX1_CP1_FZTM230144884-1A.ra

© Reported with Compound Discoverer 3.3

Specialized Traces
02-Jan-2024 11:35

Checked **Spectrum File**

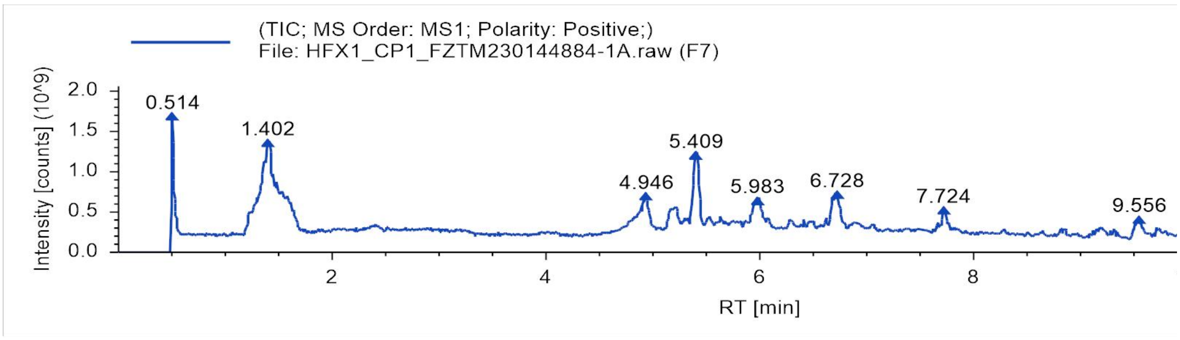

HFX1_CP1_FZTM230144885-1A.ra

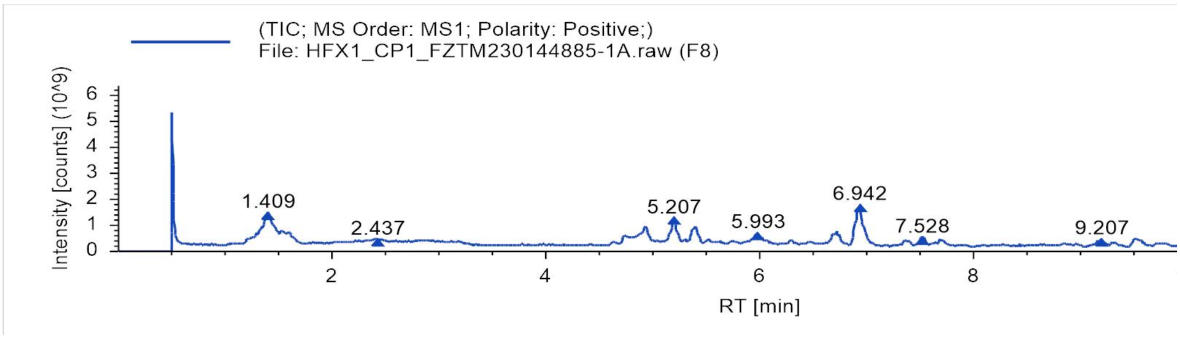

HFX1_CP1_FZTM230144886-1A.ra

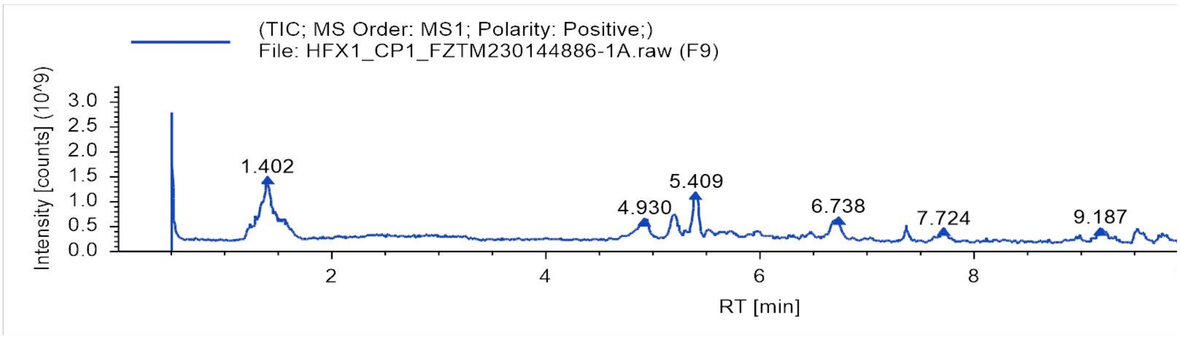

HFX1_CP1_FZTM230144887-1A.ra

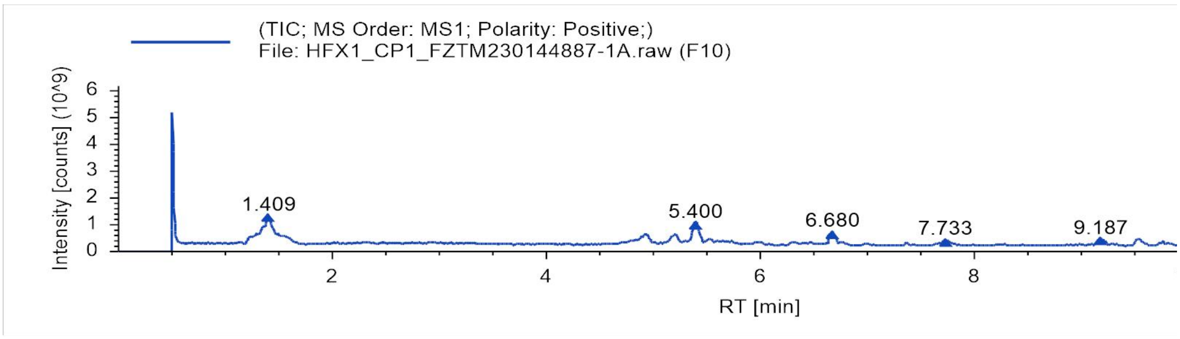

HFX1_CP2_FZTM230144888-1A.ra

Specialized Traces
02-Jan-2024 11:35

Checked **Spectrum File**

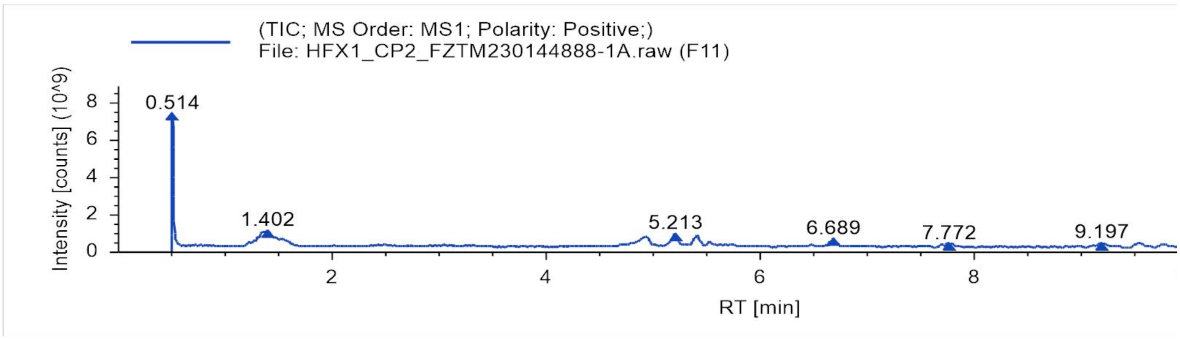

HFX1_CP2_FZTM230144889-1A.ra

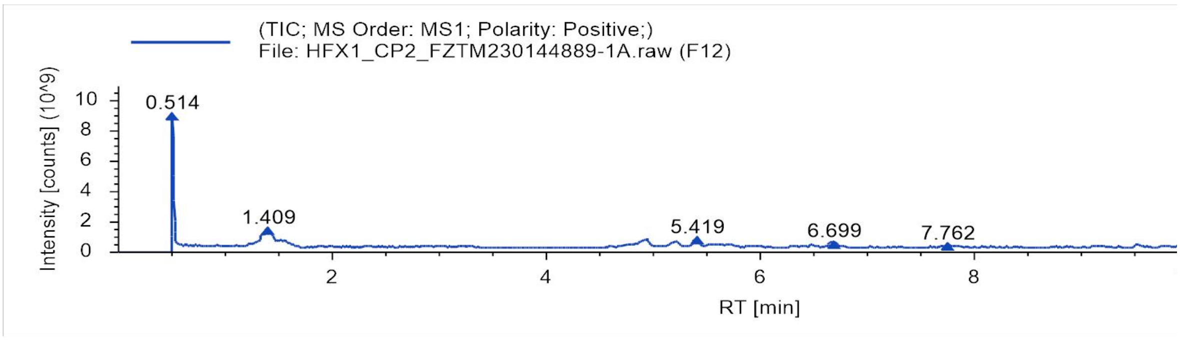

HFX1_CP2_FZTM230144890-1A.ra

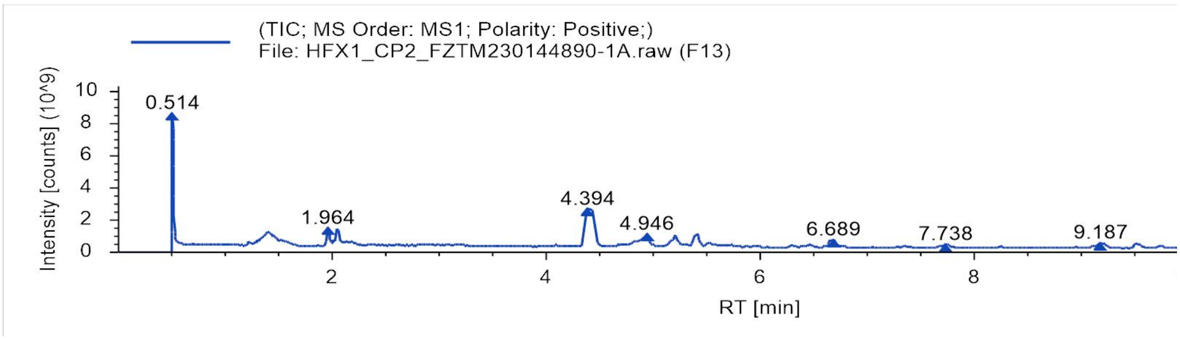

HFX1_CP2_FZTM230144891-1A.ra

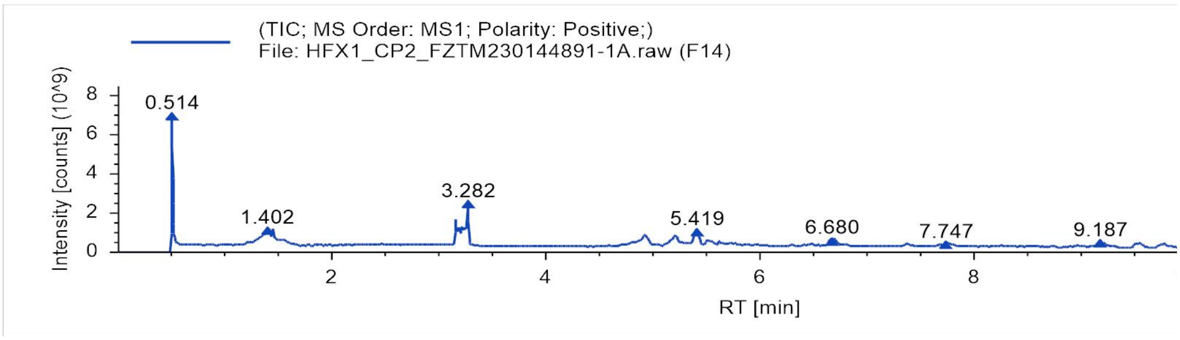

HFX1_CP2_FZTM230144892-1A.ra

© Reported with Compound Discoverer 3.3

Specialized Traces
02-Jan-2024 11:35

Checked	Spectrum File

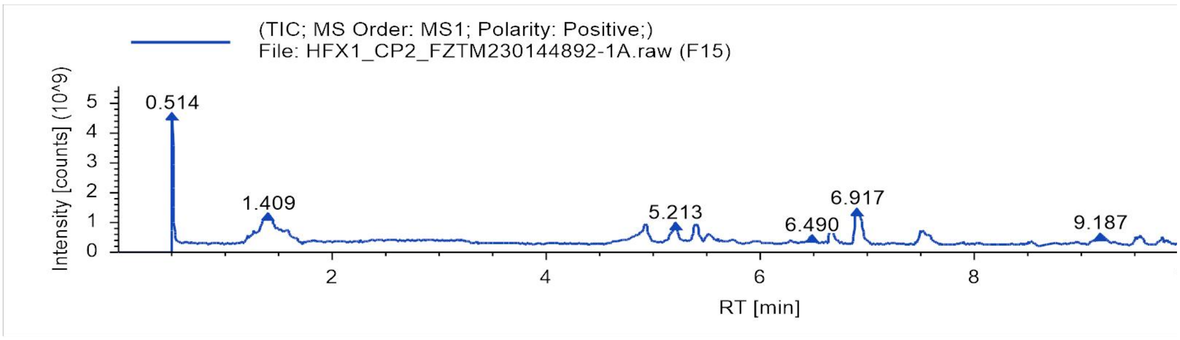

HFX1_CP2_FZTM230144893-1A.ra

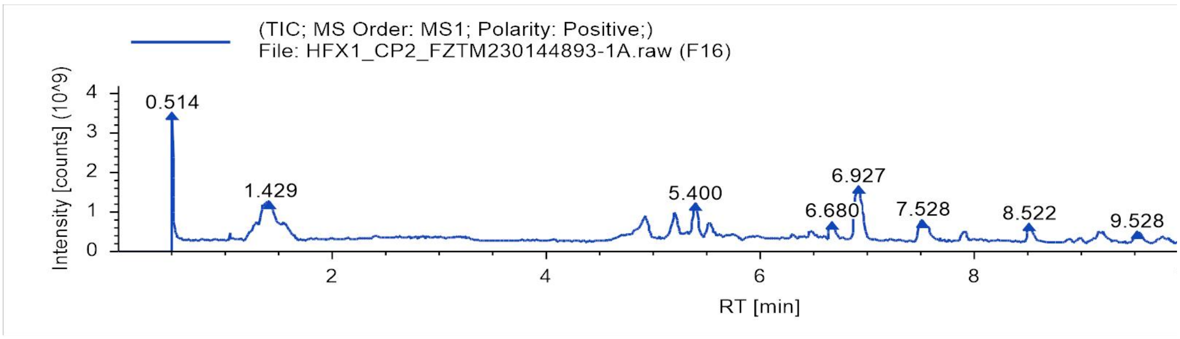

HFX1_CP3_FZTM230144894-1A.ra

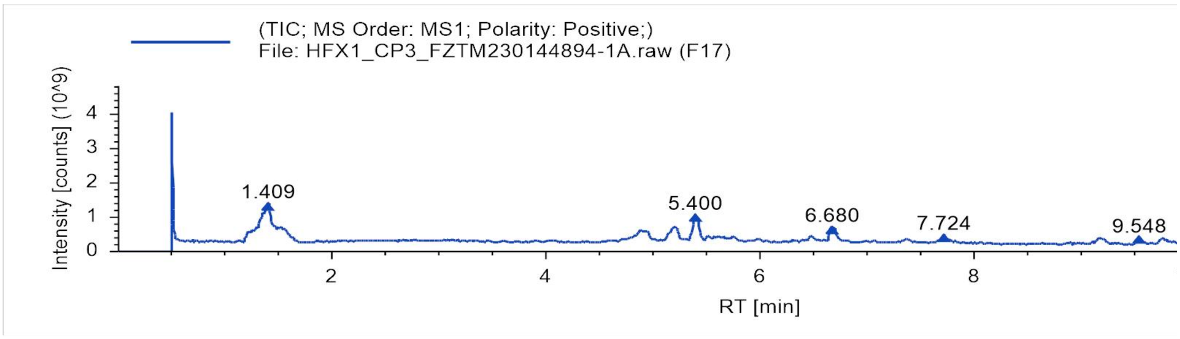

HFX1_CP3_FZTM230144895-1A.ra

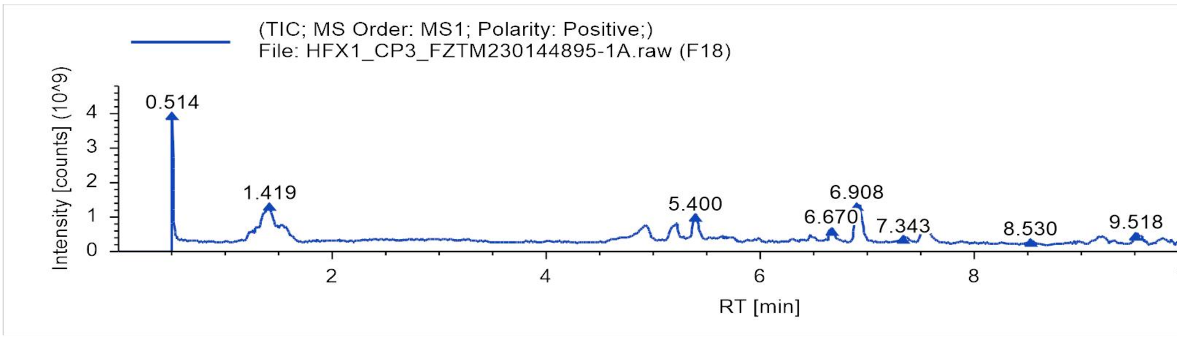

HFX1_CP3_FZTM230144896-1A.ra

© Reported with Compound Discoverer 3.3

Specialized Traces
02-Jan-2024 11:35

Checked **Spectrum File**

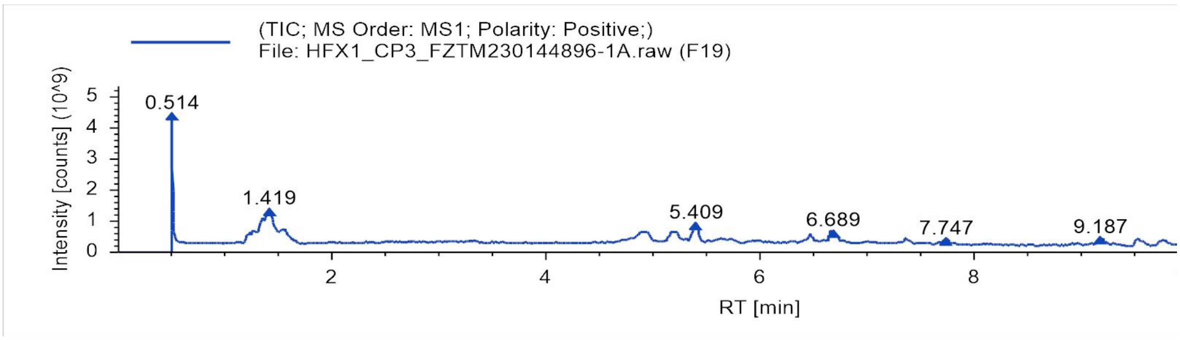

HFX1_CP3_FZTM230144897-1A.ra

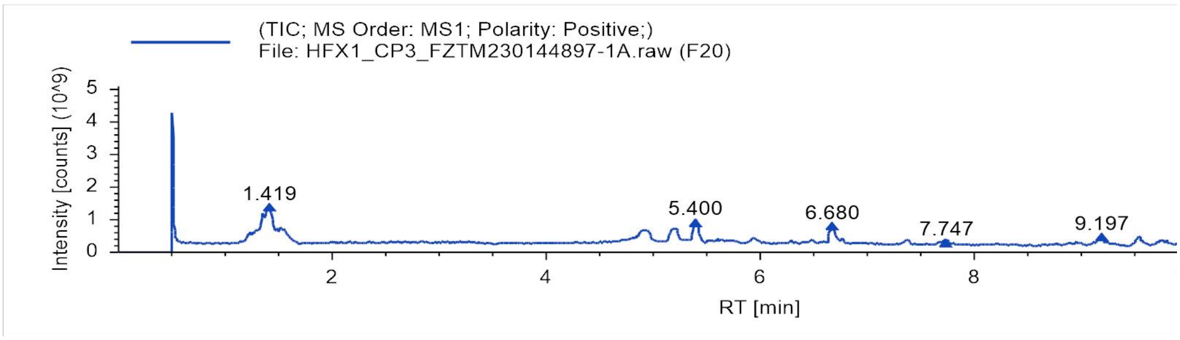

HFX1_CP3_FZTM230144898-1A.ra

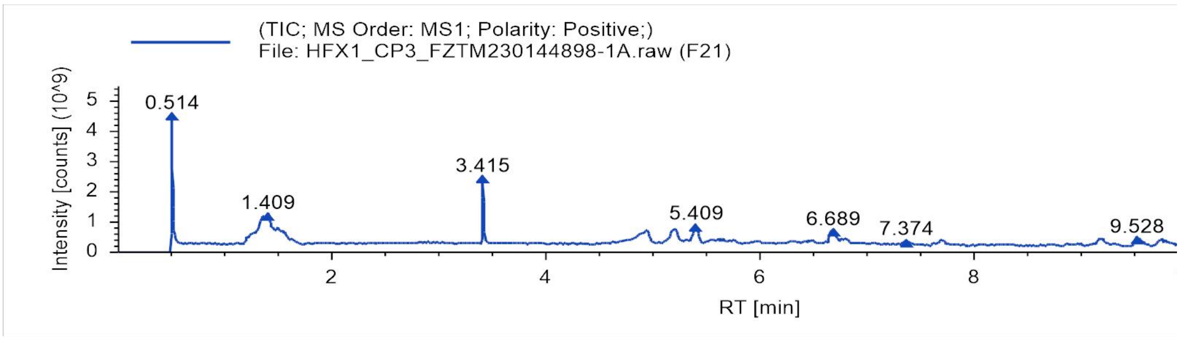

HFX1_CP3_FZTM230144899-1A.ra

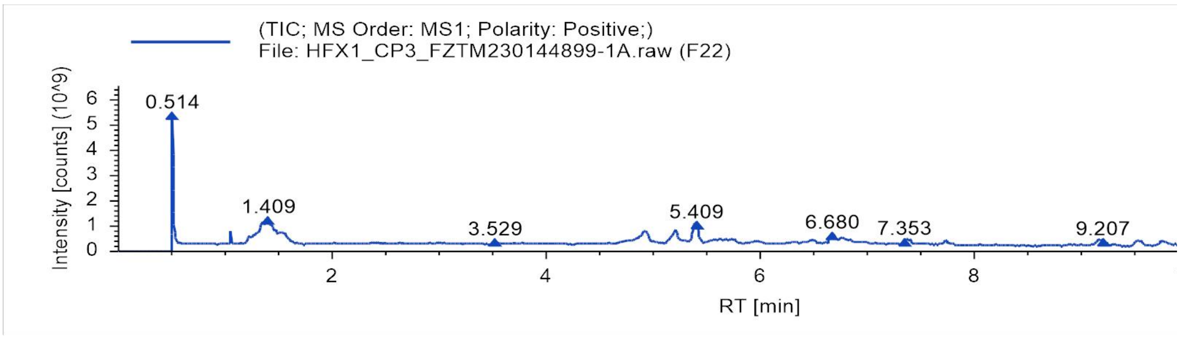

HFX1_2139130_CP_blank.raw

© Reported with Compound Discoverer 3.3

Specialized Traces
02-Jan-2024 11:35

Checked | **Spectrum File**

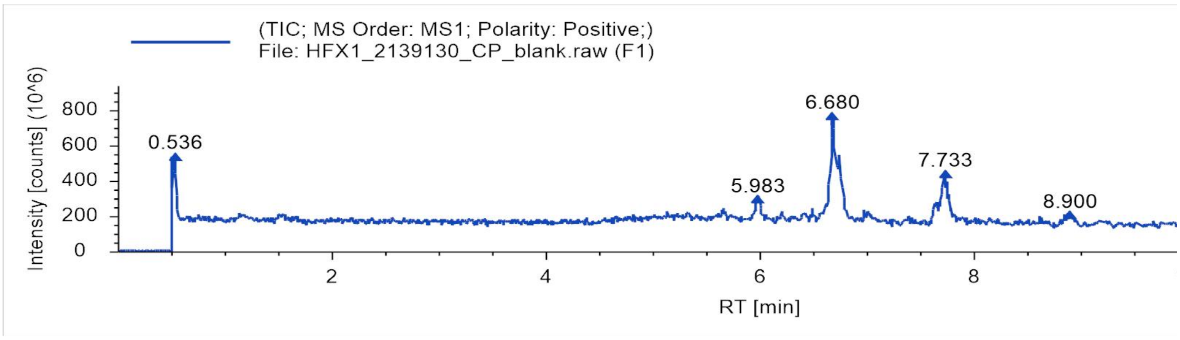

HFX1_2139130_CP_QC1.raw

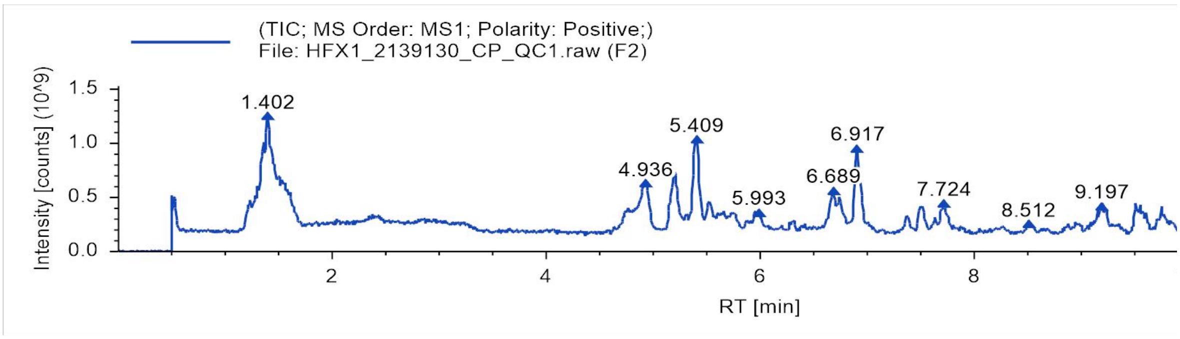

© Reported with Compound Discoverer 3.3

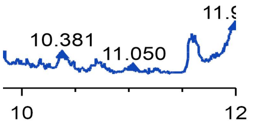

10.381
11.050
11.9
10
12

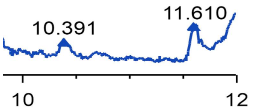

10.391
11.610
10
12

ıW

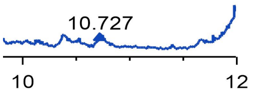

10.727
10
12

ıW

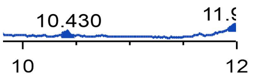

10.430
11.9
10
12

ıW

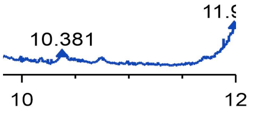

ıW

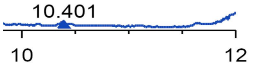

ıW

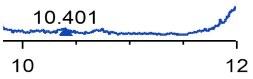

ıW

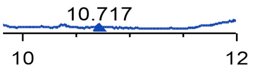

ıW

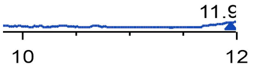

ıW

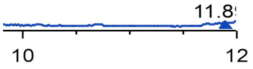

ıW

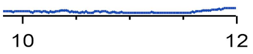

ıW

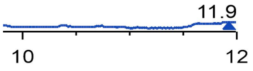

ıW

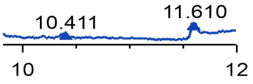
10.411
11.610
10
12

ıW

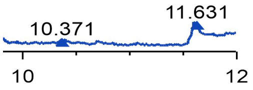
11.631
10.371
10
12

ıW

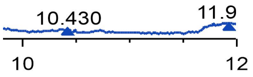
10.430
11.9
10
12

ıW

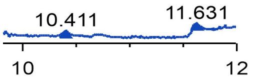
10.411
11.631
10
12

ıW

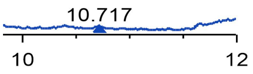

ıW

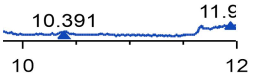

ıW

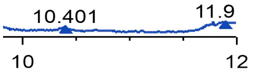

ıW

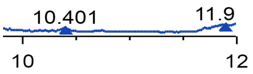

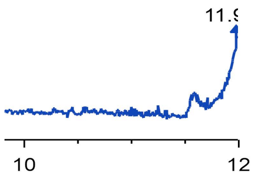
10
12

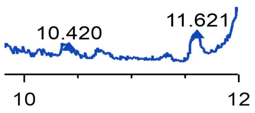
10.420
11.621
10
12
