# Supplementary material for: Research on the Changing Characteristics of Milk Composition and Serum Metabolites Across Various Lactation Periods in Xinggao Sheep
Source: Metabolites. 2025 Oct 20;15(10):678. doi: 10.3390/metabo15100678 (PMC12566154; doi:10.3390/metabo15100678)
Supplement: Supplementary file 1 [file metabolites-15-00678-s001.zip › Supplementary Material/TABLE/S Table4.pdf]

MA vs MB

Pathway Name	Match Status	p	-log(p)	Holm p	FDR	Impact
Pyrimidine metabolism	5/39	0.0021219	2.6733	0.16975	0.16975	0.13999
Glycerophospholipid metabolism	4/36	0.010308	1.9868	0.81435	0.41233	0.1699
Arachidonic acid metabolism	4/44	0.020637	1.6854	1.0	0.55032	0.46192
Sphingolipid metabolism	3/32	0.041291	1.3841	1.0	0.59553	0.15592
Arginine biosynthesis	2/14	0.044207	1.3545	1.0	0.59553	0.12234
Glycine, serine and threonine metabolism	3/33	0.044665	1.35	1.0	0.59553	0.0
Arginine and proline metabolism	3/36	0.055589	1.255	1.0	0.63531	0.04535
Purine metabolism	4/70	0.088527	1.0529	1.0	0.75627	0.03064
Phenylalanine, tyrosine and tryptophan biosynthesis	1/4	0.094533	1.0244	1.0	0.75627	0.5
Riboflavin metabolism	1/4	0.094533	1.0244	1.0	0.75627	0.5
Linoleic acid metabolism	1/5	0.11677	0.93267	1.0	0.84851	0.0
One carbon pool by folate	2/26	0.13156	0.88087	1.0	0.84851	0.04489
Nitrogen metabolism	1/6	0.13848	0.85863	1.0	0.84851	0.0
Alanine, aspartate and glutamate metabolism	2/28	0.14849	0.8283	1.0	0.84851	0.28366
Phenylalanine metabolism	1/8	0.18034	0.74391	1.0	0.8912	0.0
Glyoxylate and dicarboxylate metabolism	2/32	0.18355	0.73625	1.0	0.8912	0.03
Cysteine and methionine metabolism	2/33	0.19251	0.71555	1.0	0.8912	0.19943
Vitamin B6 metabolism	1/9	0.20052	0.69784	1.0	0.8912	0.07843
Biosynthesis of unsaturated fatty acids	2/36	0.21972	0.65812	1.0	0.92515	0.0
Tryptophan metabolism	2/41	0.26574	0.57554	1.0	1.0	0.13228

MA vs MC

Pathway Name	Match Status	p	-log(p)	Holm p	FDR
Linoleic acid metabolism	2/5	0.005031	2.2983	0.40248	0.34194
Glycerophospholipid metabolism	4/36	0.0085486	2.0681	0.67534	0.34194
Steroid hormone biosynthesis	6/87	0.013229	1.8785	1.0	0.35277
One carbon pool by folate	3/26	0.0208	1.6819	1.0	0.416
Tryptophan metabolism	3/41	0.06724	1.1724	1.0	0.89856
Arachidonic acid metabolism	3/44	0.079689	1.0986	1.0	0.89856
Phenylalanine, tyrosine and tryptophan biosynthesis	1/4	0.089856	1.0465	1.0	0.89856
Riboflavin metabolism	1/4	0.089856	1.0465	1.0	0.89856
Phenylalanine metabolism	1/8	0.17184	0.76489	1.0	1.0
Glycine, serine and threonine metabolism	2/33	0.1774	0.75106	1.0	1.0
Biosynthesis of unsaturated fatty acids	2/36	0.20293	0.69266	1.0	1.0
Arginine and proline metabolism	2/36	0.20293	0.69266	1.0	1.0
Biotin metabolism	1/10	0.21009	0.6776	1.0	1.0
Purine metabolism	3/70	0.22004	0.65751	1.0	1.0
Tyrosine metabolism	2/42	0.25508	0.59332	1.0	1.0
alpha-Linolenic acid metabolism	1/13	0.26426	0.57797	1.0	1.0
Arginine biosynthesis	1/14	0.2815	0.55053	1.0	1.0
D-Amino acid metabolism	1/15	0.29834	0.52528	1.0	1.0
Nicotinate and nicotinamide metabolism	1/15	0.29834	0.52528	1.0	1.0
Glycerolipid metabolism	1/16	0.31481	0.50196	1.0	1.0

MB vs MC

Pathway Name	Match Status	p	-log(p)	Holm p	FDR	Impact
Arginine biosynthesis	4/14	2.097E-4	3.6784	0.016776	0.016776	0.31915
Arachidonic acid metabolism	5/44	0.002886	2.5397	0.22799	0.11544	0.0695
Steroid hormone biosynthesis	6/87	0.013229	1.8785	1.0	0.35277	0.12927
Pyrimidine metabolism	3/39	0.059492	1.2255	1.0	1.0	0.09576
Tyrosine metabolism	3/42	0.071281	1.147	1.0	1.0	0.05427
Riboflavin metabolism	1/4	0.089856	1.0465	1.0	1.0	0.5
Linoleic acid metabolism	1/5	0.11106	0.95444	1.0	1.0	0.0
One carbon pool by folate	2/26	0.12058	0.91871	1.0	1.0	0.13264
Glutathione metabolism	2/28	0.13631	0.86547	1.0	1.0	0.03001
Cysteine and methionine metabolism	2/33	0.1774	0.75106	1.0	1.0	0.12535
Vitamin B6 metabolism	1/9	0.19118	0.71856	1.0	1.0	0.0
Glycerophospholipid metabolism	2/36	0.20293	0.69266	1.0	1.0	0.11201
Arginine and proline metabolism	2/36	0.20293	0.69266	1.0	1.0	0.14535
alpha-Linolenic acid metabolism	1/13	0.26426	0.57797	1.0	1.0	0.0
D-Amino acid metabolism	1/15	0.29834	0.52528	1.0	1.0	0.0
Butanoate metabolism	1/15	0.29834	0.52528	1.0	1.0	0.0
Histidine metabolism	1/16	0.31481	0.50196	1.0	1.0	0.0
Terpenoid backbone biosynthesis	1/18	0.34661	0.46016	1.0	1.0	0.0
Pantothenate and CoA biosynthesis	1/20	0.37698	0.42368	1.0	1.0	0.0
Citrate cycle (TCA cycle)	1/20	0.37698	0.42368	1.0	1.0	0.05856
