# Supplementary figures and images for: Research on the Changing Characteristics of Milk Composition and Serum Metabolites Across Various Lactation Periods in Xinggao Sheep
Source: Metabolites. 2025 Oct 20;15(10):678. doi: 10.3390/metabo15100678 (PMC12566154; doi:10.3390/metabo15100678)

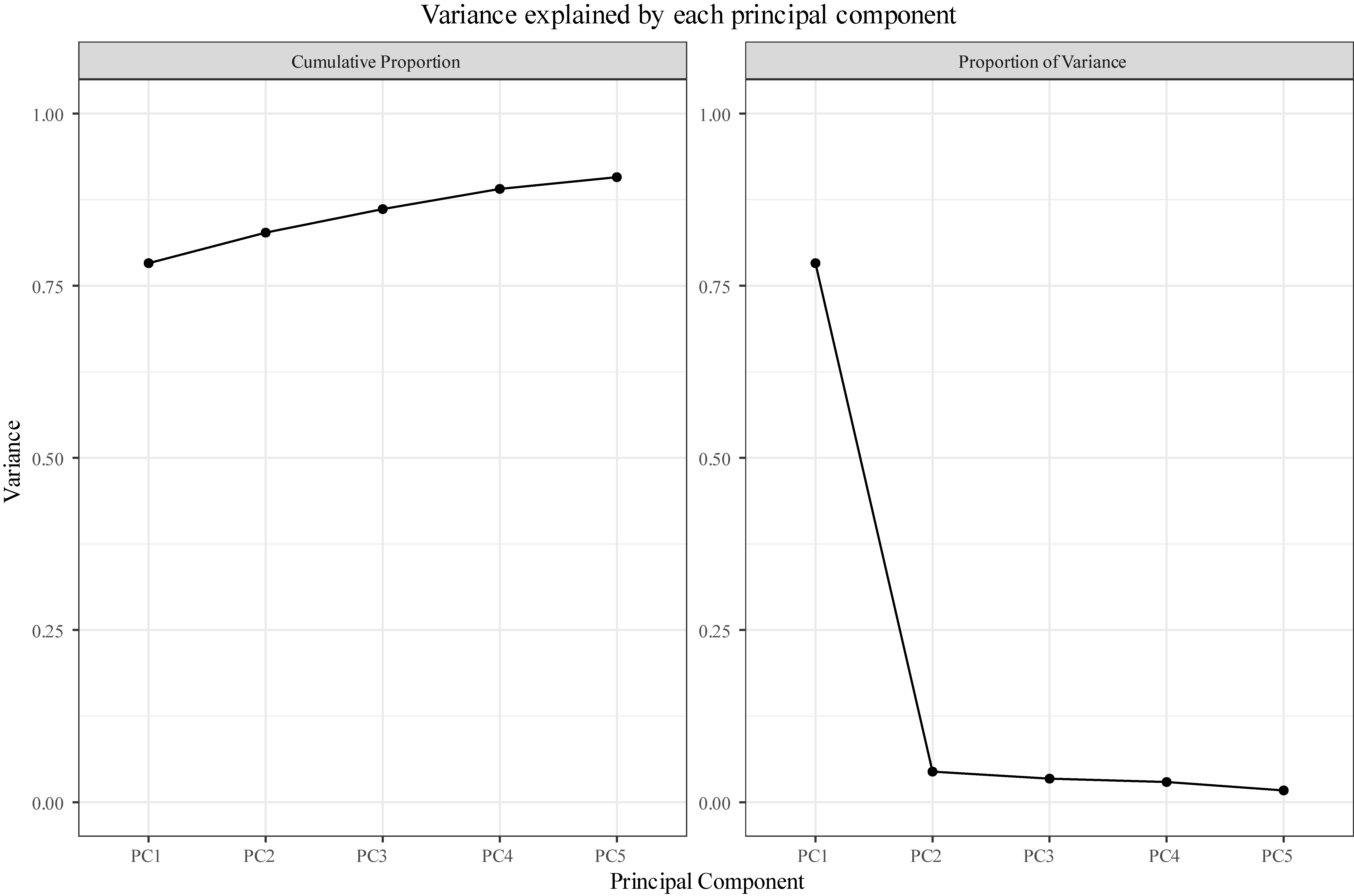

Supplement: Supplementary file 1 [file metabolites-15-00678-s001.zip › Supplementary Material/FIG/S Fig1.tif]
